# Progressive Alcohol-Related Brain Atrophy and White Matter Pathology Are Linked to Long-Term Inhibitory Effects on mTOR Signaling

**DOI:** 10.3390/biom15030413

**Published:** 2025-03-14

**Authors:** Ming Tong, Camilla Homans, William Pelit, Busra Delikkaya, Suzanne M. de la Monte

**Affiliations:** 1 Department of Medicine, Rhode Island Hospital, Brown University Health, and The Warren Alpert Medical School of Brown University, Providence, RI 02903, USA; mtong216@gmail.com; 2 Molecular Pharmacology, Physiology, and Biotechnology Graduate Program, Brown University, Providence, RI 02903, USA; 3 Department of Chemistry, Brown University, Providence, RI 02903, USA; 4 Department of Pathology and Laboratory Medicine, Rhode Island Hospital, Brown University Health, The Providence VA Medical Center, and the Warren Alpert Medical School of Brown University, Providence, RI 02903, USA; busra.delikkaya@jefferson.edu; 5 Departments of Neurosurgery and Neurology, Rhode Island Hospital, Brown University Health, and The Warren Alpert Medical School of Brown University, Providence, RI 02903, USA

**Keywords:** alcohol-related brain damage, white matter, rat model, mTOR, oligodendrocytes

## Abstract

Background: Alcohol-related brain damage (ARBD) causes cognitive-behavioral impairments that can lead to dementia. White matter is a major target in ARBD. Additional research is needed to better understand the mechanisms of ARBD progression to advanced stages with permanent disability. Potential contributing factors include neuroinflammation and altered signaling through pathways that regulate cell survival, neuronal plasticity, myelin maintenance, and energy metabolism. Objectives: This study characterizes the time course-related effects of chronic heavy ethanol feeding on white matter myelin protein expression, neuroinflammation, and molecules that mediate signaling through the mechanistic target of rapamycin (mTOR) pathways. Methods: Adult Long Evans rats (8–12/group) were fed with isocaloric liquid diets containing 0% (control) or 36% ethanol. Experimental endpoints spanned from 1 day to 8 weeks. The frontal lobes were used for histopathology and molecular and biochemical analyses. Results: Chronic ethanol feeding caused significant brain atrophy that was detected within 4 weeks and sustained over the course of the study. Early exposure time points, i.e., 2 weeks or less, were associated with global increases in the expression of non-myelinating, myelinating, and astrocyte markers, whereas at 6 or 8 weeks, white matter oligodendrocyte/myelin/glial protein expression was reduced. These effects were not associated with shifts in neuroinflammatory markers. Instead, the early stages of ARBD were accompanied by increases in several mTOR proteins and phosphoproteins, while later phases were marked by inhibition of downstream mTOR signaling through P70S6K. Conclusions: Short-term versus long-term ethanol exposures differentially altered white matter glial protein expression and signaling through mTOR’s downstream mediators that have known roles in myelin maintenance. These findings suggest that strategic targeting of mTOR signaling dysregulation may be critical for maintaining the functional integrity of white matter and ultimately preventing long-term ARBD-related cognitive impairment.

## 1. Introduction

Alcohol use disorder (AUD) compromises health status, family dynamics, economic security, and social well-being [1,2,3], and with moderate to high degrees of severity, it can substantially contribute to morbidity and mortality linked to multi-organ and system dysfunction [4]. Chronic, heavy alcohol misuse adversely impacts the brain, causing atrophy, cognitive impairment with deficits in executive function and memory, and in advanced cases, dementia and disability [5,6]. In the central nervous system (CNS), chronic, sustained alcohol-related neurobehavioral pathologies are mediated by neurotoxic and degenerative effects in white matter, particularly in prefrontal regions, the temporal lobes, cerebellum, and corpus callosum [7,8]. Higher doses and longer durations of chronic or binge alcohol consumption correlate with the severity of CNS damage from alcohol misuse [6,9,10]

Alcohol’s neurotoxic and neurodegenerative effects target CNS white matter myelin and axons [6,8,10]. Oligodendrocytes, which generate and maintain myelin [11,12], are highly susceptibility to alcohol’s neurotoxic and degenerative effects [13,14]. Mechanistically, moderate to heavy ethanol exposures impair signaling pathways stemming from the insulin and IGF-1 receptors and directed downstream to support critical oligodendrocyte functions, including cell survival, energy metabolism, and myelin homeostasis [15,16,17]. Previous studies showed that ethanol’s inhibitory effects on phosphatidylinositol-3-kinase (PI3K)-Akt and attendant activation of glycogen synthase kinase-3β (GSK-3β) compromise cell survival and cytoprotective defenses against oxidative injury, DNA damage, and disrupted energy balance. However, those adverse effects of ethanol were largely observed in developing and adolescent rather than adult models [18,19,20]. Correspondingly, there is ample evidence that immature developing brains are substantially more sensitive to the neurotoxic and long-term damaging effects of alcohol compared with mature adult brains [21,22]. Mechanistically, the prominent inhibitory effects of ethanol on insulin/IGF-PI3K-Akt signaling in the immature brain likely account for cell loss, impaired cell growth, survival, and metabolic functions needed for cognitive-behavioral functions. Such adverse effects in the immature brain contribute to the long-lasting cognitive-behavioral dysfunctions that persist through later periods in life [23,24,25,26].

An additional intracellular signaling network that is adversely affected by ethanol in the brain is linked to mechanistic target of rapamycin (mTOR) [27,28,29]. The mTOR pathways have important roles in oligodendrocyte functions such myelin formation, maintenance, and integrity [30,31]. Signaling through the Rapamycin-sensitive mTOR protein complex 1 (mTORC1) [32,33], which includes the Raptor adaptor protein [33], stimulates myelin production by oligodendrocytes. Correspondingly, rapamycin inhibition of mTOR/mTORC1 impairs oligodendrocyte survival and maturation, myelin synthesis, and myelin maintenance, resulting in white matter loss or atrophy [30,31,34]. mTORC2, which is rapamycin-insensitive, includes the Rictor adaptor protein [33] and modulates metabolic signaling through P70S6K [35]. Compromised signaling through mTOR/mTORC2 impairs cellular metabolism via its inhibitory effects on ^S473^-Akt (Akt activation) and p-P70S6K [30,31,34]. Altogether, these findings highlight the importance of mTOR/mTORC1/mTORC2 signaling in relation to oligodendrocyte function and myelin homeostasis [30,31,35,36] and justify further study in relation to ARBD in the adult brain.

It has been well established that high-dose chronic alcohol exposures cause sustained neurobehavioral dysfunctions, neuropathology, and molecular and biochemical impairments in the CNS [20,37] and that the severity of ARBD increases with lifetime duration and maximum exposure [38,39,40]. Although evidence suggests that ARBD can be partially reversed by abstinence [41,42,43,44,45], the pathophysiological factors that drive its progression and sustain chronicity have not been determined. To address this problem, we utilized an established adult model of chronic heavy alcohol consumption to measure time course-related molecular and biochemical changes leading ARBD. The investigations focused on white matter myelin/oligodendrocyte pathology, indices of neuroinflammation, and mTOR signal transduction pathways.

## 2. Materials and Methods

Materials: The reagents and critical instruments/technology used for this research are listed in Supplementary Table S1. The commercial antibodies and their sources, final concentrations, vendors, catalog numbers, and Research Resource Identifiers (RRID) are listed in Supplementary Table S2.

Experimental Model: This study examined the time course effects of high-dose chronic ethanol feeding in an established adult Long Evans rat model. Six-week-old male and female rats (n = 8–12/group) (Charles River Laboratories, Wilmington, MA USA) were pair-fed with Lieber-DeCarli isocaloric liquid diets (BioServ, Frenchtown, NJ, USA) that contained 0% or 36% (caloric) ethanol for periods ranging from 1 day to 8 weeks (56 days) following four days of liquid diet adaptation. Food intake and body weight were monitored regularly. The rats were housed in same-sex pair cages in a pathogen-free animal facility with an automated 12 h light/dark cycle (lights on at 7:00 a.m. and off at 7:00 p.m.).

Experimental Endpoint Procedures: At the experimental endpoints, between 9:00 a.m. and 12:00 p.m., blood ethanol concentrations were measured using a colorimetric assay kit. The rats were then euthanized under deep isoflurane anesthesia by cardiac puncture exsanguination. The brains and livers were weighed. The brains were immediately sectioned to obtain 3 mm coronal plane slices of the frontal lobes from just anterior to the temporal poles. One frontal lobe from each rat, including cortical ribbon and underlying white matter, was snap-frozen on dry ice and preserved in an air-tight container at −80 °C for later molecular assays. The contralateral frontal lobe was immersion-fixed in formalin and embedded in paraffin. Histological sections were stained with Luxol fast blue, hematoxylin and eosin (LHE) to assess white matter myelin integrity.

Protein Homogenates: Using a TissueLyser II (Qiagen, Germantown, MD, USA) with 5 mm stainless steel beads, the brain tissue samples were homogenized in weak lysis buffer that contained protease and phosphatase inhibitors [46]. Supernatant fractions clarified by centrifugation at 14,000× *g* for 10 min were aliquoted for enzyme-linked immunosorbent assays (ELISAs). Protein concentrations were measured with the bicinchoninic (BCA) assay.

Duplex ELISAs: These assays measured immunoreactivity to white matter oligodendrocyte, myelin, or astrocyte proteins, including 2′3′-cyclic nucleotide 3′-phosphodiesterase (CNPase), myelin proteolytic protein (PLP), platelet-derived growth factor receptor-alpha (PDGFRA), Galactosylceramidase (GalC), myelin-associated glycoprotein 1 (MAG1), myelin oligodendrocyte glycoprotein (MOG), myelin basic protein (MBP), Nestin, Vimentin, and glial fibrillary acidic protein (GFAP) (Supplementary Table S2). In addition, immunoreactivity to the mTOR-related signaling molecules Rictor, Raptor, ^S1591^p-Rictor, and ^S792^p-Raptor was measured by duplex ELISA. In brief, triplicate 50 ng protein aliquots were adsorbed to the bottom surfaces of 96-well MaxiSorp plates overnight at 4 °C. Superblock (TBS) was used to mask non-specific binding sites. Immunoreactivity was detected by a 4 °C overnight incubation with primary antibody, followed by sequential horseradish peroxidase-conjugated secondary antibody and Amplex UltraRed incubations. Fluorescence intensity was measured in a SpectraMax M5 microplate reader (excitation 530 nm/emission 590 nm). Large acidic ribosomal protein (RPLPO) immunoreactivity was detected with biotinylated anti-RPLPO followed by streptavidin-conjugated alkaline phosphatase, and 4-MUP (Ex360 nm/Em450 nm) served as a loading control [47,48,49]. The calculated ratios of the specific protein to RPLPO fluorescence were used for intergroup statistical comparisons.

Multiplex ELISAs: Magnetic bead-based total and phospho-Akt/mTOR 11-Plex panels were used to examine the effects of ethanol exposure and duration on the expression and phosphorylation of proteins integrally related to insulin and IGF-1 signaling through the Akt and mTOR pathways (Supplementary Table S3A). The functions of the assayed proteins and phosphoproteins are summarized in Supplementary Table S4. Immunoreactivity was measured in 12.5 μg protein aliquots of frontal lobe homogenates. A 5-plex magnetic bead-based cytokine assay (Supplementary Table S3B) measured proinflammatory cytokines in 150 µg protein sample aliquots. The multiplex ELISAs were performed according to the manufacturer’s protocols. In brief, following incubation with antibody-bound beads, fluorescence intensity, corresponding to captured antigens detected with biotinylated secondary antibodies and Streptavidin-conjugated phycoerythrin-conjugated streptavidin, was measured in a MAGPIX. Results were analyzed using xPONENT software (xPONENT 4.3, Luminex Corp, Austin, TX, USA). Standard curves for each analyte were included to calculate the levels of immunoreactivity. Results are expressed as fluorescent light units (FLU) or pmol/150 μg protein).

Statistics: Results were analyzed by two-way mixed-model analysis of variance (ANOVA) with post hoc Tukey multiple comparisons tests (GraphPad Prism 10.4, San Diego, CA, USA) to examine the effects of chronic progressive heavy ethanol exposure on white matter molecules, Akt-mTOR signaling, and neuroinflammation. The F-ratios and *p*-values are shown in Table 1, Table 2, Table 3, Table 4 and Table 5, with significant (*p* ≤ 0.05) or trend-wise (0.05 < *p* < 0.10) differences highlighted. Significant post hoc test results are shown in the graph panels. Heatmaps and bar plots were used to display the within-group and between-group differences in marker expression measured by ELISA.

## 3. Results

**Figure 1:**
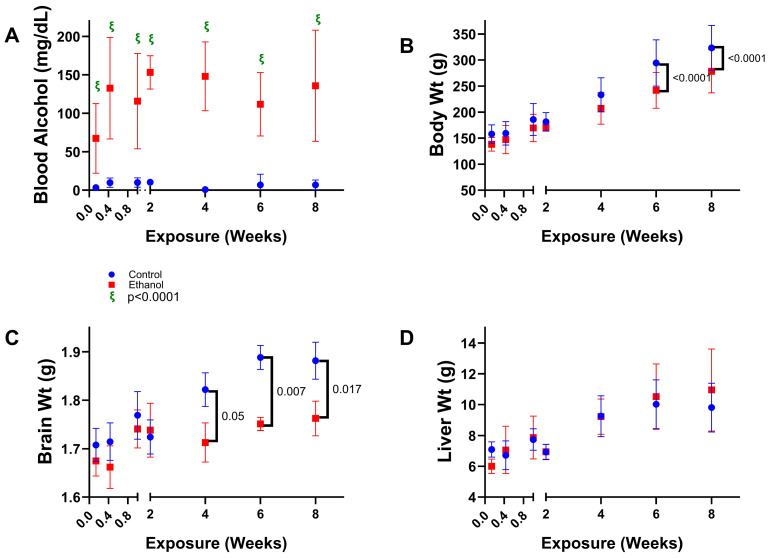
Effects of chronic high-dose ethanol exposures on blood alcohol, body weight, brain weight, and liver weight. Long Evans male and female rats were fed with isocaloric liquid diets containing 0% (control) or 36% ethanol (n = 8–12/group) from 1 day to 56 days after a 4-day period of adaptation. Terminal (experimental endpoint) mean (±S.D.) of (**A**) blood alcohol levels, (**B**) body weights, (**C**) brain weights, and (**D**) liver weights were compared between the groups by two-way ANOVA (See Table 1) with post hoc Tukey tests. Significant differences correspond to *p* ≤ 0.05. Statistical trend-wise differences (italicized) reflect 0.10 < *p* < 0.05.

General Characteristics: Two-way ANOVA tests demonstrated significant ethanol effects on blood alcohol concentration (BAC), body weight, and brain weight, significant exposure duration effects on body weight, brain weight, and liver weight, and a statistical trend-wise effect of exposure duration on BAC (Table 1). Significant or trend-wise exposure × duration interactive effects were not detected. Graphs corresponding to the changes in BAC levels, body weight, brain weight, and liver weight are shown in Figure 1. At all experimental endpoints, the blood alcohol concentrations were significantly elevated in ethanol-fed rats (Figure 1A). However, the smallest difference from control occurred after one day of 36% ethanol feeding and the largest was between the 1- and 2-week experimental endpoints. Body weight increased progressively in both control and ethanol-fed rats (Figure 1B). Within the initial 2 weeks of liquid diet feeding, the mean body weights were similar for the two groups, but from 4 weeks and beyond, ethanol feeding resulted in progressively smaller weight gains resulting in significant inter-group differences at the 6- and 8-week time points. Daily monitoring revealed that control rats consistently consumed 100% of the food provided twice daily over the time course. In contrast, the ethanol-fed rats consumed just 90% to 95% of the food during the first two weeks (leaving 5–10%), but subsequently they also consumed all the twice-daily-supplied food. Since the rats were housed in pairs, it was not possible to assess differences in food consumption. On the other hand, the rats did not lose weight and the weekly percentage weight gains in cage mates differed by less than 10%. During the initial two weeks of the study, there were no significant effects of ethanol on mean brain weight. However, after 4, 6, and 8 weeks of ethanol feeding, significant ethanol-associated reductions in mean brain weight were observed (Figure 1C). Liver weights gradually and similarly increased in the control and ethanol-diet groups with no significant differences measured over the duration of the study (Figure 1D).

**Table 1:** Model characteristics—two-way ANOVA tests.

Feature	Ethanol F Ratio	*p*-Value	Duration F Ratio	*p*-Value	Ethanol × Duration F Ratio	*p*-Value
**Blood Alcohol**	**196.3**	**<0.0001**	*1.90*	*0.09*	1.639	N.S.
**Body Weight**	**18.56**	**<0.0001**	**57.05**	**<0.0001**	1.135	N.S.
**Brain Weight**	**10.44**	**0.0018**	**4.527**	**0.0005**	1.001	N.S.
**Liver Weight**	0.247	N.S.	**18.54**	**<0.0001**	0.767	N.S.

Two-way ANOVA tests comparing the effects of ethanol, exposure duration (1 to 56 days), and ethanol × exposure duration interactions. Bold font highlights significant results (*p* ≤ 0.05). Italicized values reflect statistical trend effects (0.10 < *p* < 0.05). N.S. = not statistically significant. See Figure 1 for post hoc Tukey test results.

**Figure 2:**
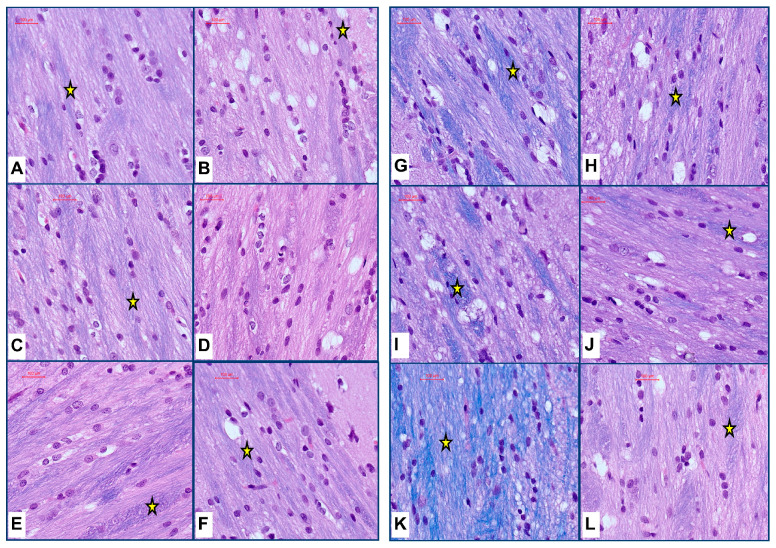
Chronic ethanol exposure reduces white matter myelin. Formalin-fixed, paraffin-embedded histological sections (5 µm thick) of the frontal lobe from control (**A**,**C**,**E**,**G**,**I**,**K**) and ethanol-fed (**B**,**D**,**F**,**H**,**J**,**L**) adult Long Evans rats were stained with LHE. The images depict representative examples of corpus callosum white matter from brains harvested on experimental endpoint Days 3 (**A**,**B**), 7 (**C**,**D**), 14 (**E**,**F**), 28 (**G**,**H**), 42 (**I**,**J**), and 56 (**K**,**L**). Luxol fast blue dye stains myelin blue (asterisks). (**B**,**D**,**F**). Short-term ethanol exposure (14 days of fewer) resulted in modest degrees of myelin staining pallor. (**H**,**J**,**L**) Ethanol exposures for 4, 6, or 8 weeks conspicuously reduced myelin staining relative to (**G**,**I**,**K**) corresponding controls. Original magnifications, 400×; Scale bars correspond to 100 µm.

Brain histopathology: LHE-stained histological sections of frontal lobe at the level of the anterior corpus callosum and caudoputamen nucleus [50] were examined and photographed by light microscopy to evaluate progressive changes in white matter myelin staining as an index of myelin integrity. Over the time course, brains from ethanol-fed rats consistently exhibited lower intensities of Luxol fast blue myelin staining in corpus callosum white matter (Figure 2). The most pronounced reductions in myelin staining were evident at the 8-week endpoint of the study (Figure 2K,L). In addition, 6 to 8 weeks of ethanol exposure were associated with subtle alterations in the glial cell morphology, manifested by a less conspicuous presence of small round condensed nuclei characteristic of oligodendrocytes (Figure 2I–L).

CNS Glial Markers: The analyses were focused on glial oligodendrocyte-myelin and astrocyte protein expression to address the role of progressive white matter pathology as a mediator of brain atrophy in ARBD. The study design was set to characterize alterations in molecular white matter pathology linked to the duration of heavy ethanol feeding. Using duplex ELISAs with the levels of specific immunoreactivity normalized to RPLPO, we measured the expression of pre- or immature myelinating proteins (CNPase, PLP, PDGFA, and GalC), myelinating proteins (MAG1, MOG, and MBP), and astrocyte/glial markers (Nestin, vimentin, and GFAP). Two-way ANOVA tests detected statistically significant effects of exposure duration for all glial markers and ethanol × exposure duration interactive effects for all markers except CNPase and Nestin (Table 2). Ethanol exposure alone accounted for significant variance with respect to MBP and a trend effect for GalC. No significant effects related to RPLPO expression were observed.

**Table 2:** Glial proteins—two-way ANOVA tests.

Molecule	Ethanol F Ratio	*p*-Value	Duration F Ratio	*p*-Value	Ethanol × Duration F Ratio	*p*-Value
**CNPase**	0.719	N.S.	**8.620**	**<0.0001**	1.65	N.S.
**PLP**	0.787	N.S.	**15.46**	**<0.0001**	**3.578**	**0.0038**
**PDGFRA**	0.893	N.S.	**5.880**	**<0.0001**	**4.84**	**0.0003**
**GalC**	*2.885*	*0.093*	**26.81**	**<0.0001**	**2.772**	**0.0178**
**MAG1**	0.593	N.S.	**22.63**	**<0.0001**	**2.757**	**0.0183**
**MOG**	2.001	N.S.	**5.228**	**0.0002**	**3.798**	**0.0025**
**MBP**	**4.011**	**0.049**	**20.41**	**<0.0001**	**3.303**	**0.0064**
**Nestin**	0.262	N.S.	**22.74**	**<0.0001**	1.252	N.S.
**Vimentin**	0.838	N.S.	**16.10**	**<0.0001**	**2.861**	**0.015**
**GFAP**	0.0352	N.S.	**63.87**	**<0.0001**	**2.911**	**0.014**
**RPLPO**	0.0305	N.S.	**0.797**	**N.S.**	**0.427**	**N.S.**

**Figure 3:**
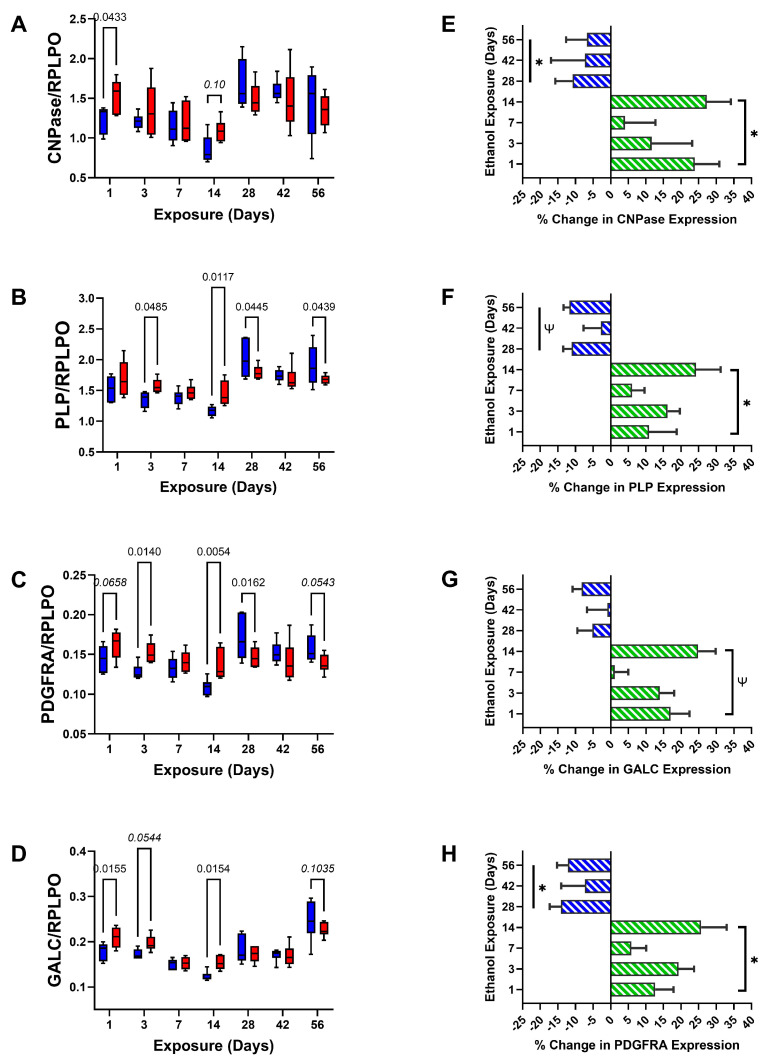
Non-myelinating/pre-myelinating glial proteins. Progressive time course-dependent changes in (**A**) CNPase, (**B**) PLP, (**C**) PDGFRA, and (**D**) GALC were measured in control (blue) and ethanol-exposed (red) frontal lobe tissue by duplex ELISA with results normalized to RPLPO. Data were analyzed by two-way ANOVA (Table 2). Significant (*p* ≤ 0.05) and statistical trend-wise differences (italicized; 0.10 < *p* < 0.05) are shown within the panels. (**E**–**H**) The calculated mean percentage changes in immunoreactivity measured in the ethanol versus corresponding control groups are depicted with bar plots. The early phase (1–14 days) stimulatory (green) versus late phase (4–8 weeks) inhibitory (blue) effects of ethanol were analyzed by one-sample *t*-tests for the null hypothesis that the percentage change = 0 (Table 3). Significant (*; *p* ≤ 0.05) and statistical trend-wise group differences (Ψ; 0.10 < *p* < 0.05) are shown within the panels.

**Figure 4:**
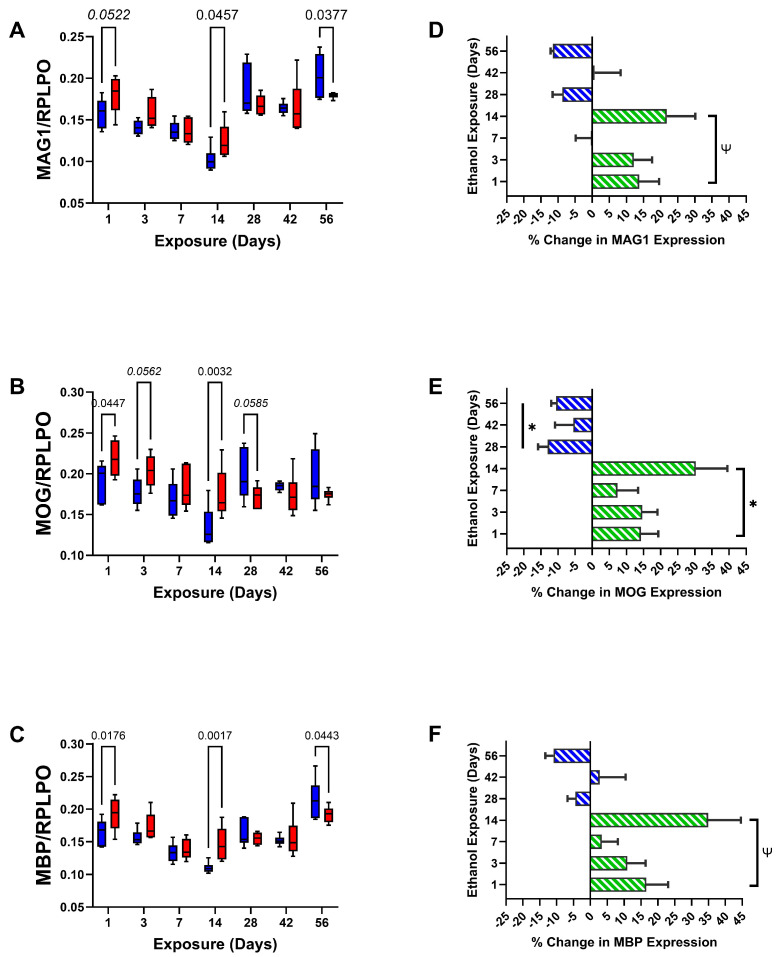
Myelinating glial proteins. Progressive time course-dependent changes in (**A**) MAG1, (**B**) MOG, and (**C**) MBP were measured in frontal lobe tissue from control (blue) and ethanol-fed (red) rats by duplex ELISA with results normalized to RPLPO. Data were analyzed by two-way ANOVA (Table 2). Significant (*p* ≤ 0.05) and statistical trend-wise differences (italicized; 0.10 < *p* < 0.05) are shown within the panels. (**D**–**F**) The calculated mean percentage differences in immunoreactivity in ethanol versus control groups are shown with bar plots. The early phase (1–14 days) stimulatory (green) versus late phase (4–8 weeks) inhibitory (blue) effects of ethanol were analyzed by one-sample *t*-tests for the null hypothesis that the percentage change = 0 (Table 3). Significant (*; *p* ≤ 0.05) and statistical trend-wise group differences (Ψ; 0.10 < *p* < 0.05) are shown within the panels.

**Figure 5:**
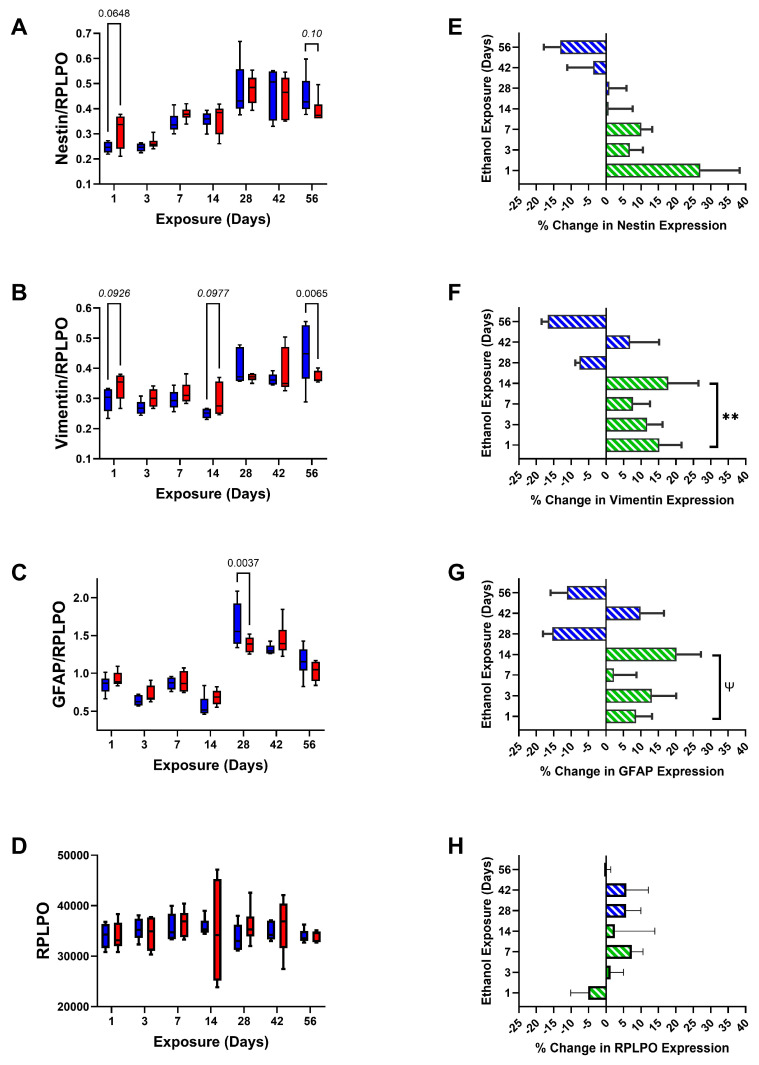
Astroglial proteins and RPLPO. Progressive changes in (**A**) Nestin, (**B**) Vimentin, and (**C**) GFAP were measured in frontal lobe tissue from control (blue) and ethanol-exposed (red) rats by duplex ELISA with results normalized to RPLPO. (**D**) RPLPO results corresponding to input protein for the ELISAs). Data were analyzed by two-way ANOVA (Table 2). Significant (*p* ≤ 0.05) and statistical trend-wise differences (italicized; 0.10 < *p* < 0.05) are shown within the panels. (**E**–**H**) The calculated mean percentage differences in immunoreactivity in ethanol versus control groups are shown with bar plots. The early-phase (1–14 days) stimulatory (green) versus late-phase (4–8 weeks) inhibitory (blue) effects of ethanol were analyzed by one-sample *t*-tests for the null hypothesis that the percentage change = 0 (Table 3). Significant (** *p* < 0.01) and statistical trend-wise group differences (Ψ; 0.10 < *p* < 0.05) are shown within the panels.

Duplex ELISASs measured glial protein immunoreactivity in frontal lobe tissue from rats maintained on isocaloric control or ethanol-containing liquid diets over a time course from 1 day to 8 weeks (n = 8/time point/group). Data were analyzed by two-way ANOVA to examine effects of ethanol exposure, duration of exposure and ethanol × duration interactive effects. Significant differences (*p* ≤ 0.05) are highlighted with bold font. Statistical trend results (0.05 < *p* < 0.10) are italicized. N.S. = not significant. Corresponding graphs with post hoc test results are displayed in Figure 3, Figure 4 and Figure 5.

**Figure 6:**
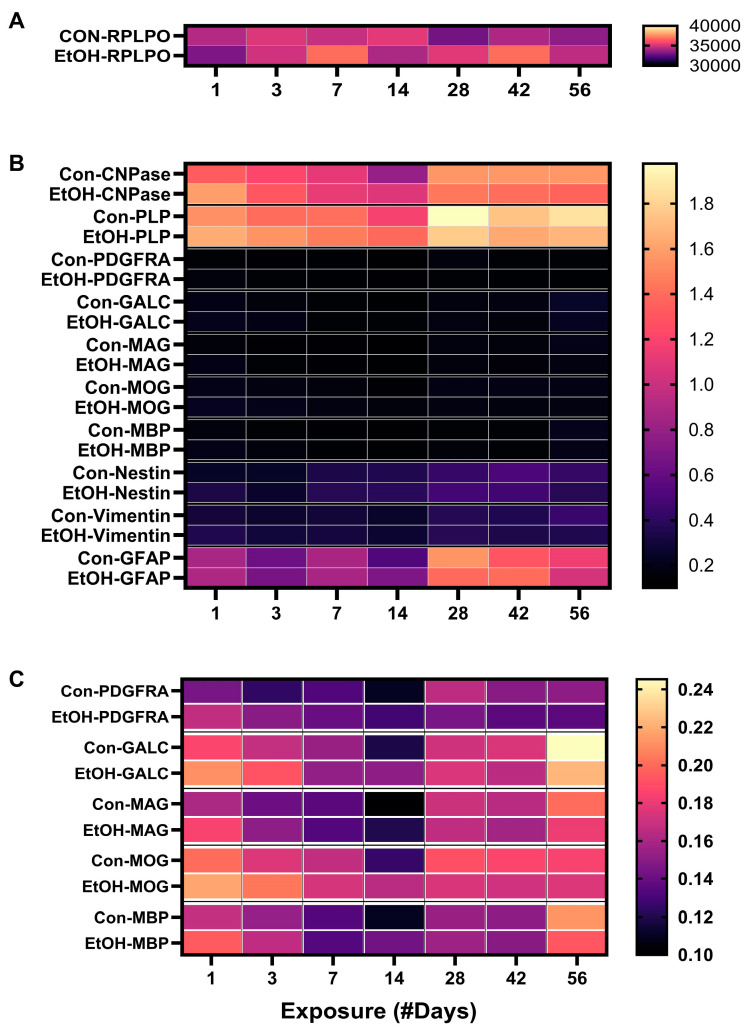
Glial protein expression heatmap. Heatmap displays of the progressive within-group and between-group time course- and ethanol exposure-related shifts levels of frontal lobe (**A**) RPLPO, (**B**) all oligodendrocyte premyelinating, oligodendrocyte myelinating, and astroglial proteins, and (**C**) the subset of relatively low-abundance oligodendrocyte/myelin proteins, PDGFA, GALC, MAG1, MOG, and MBP. Heatmap C better illustrates within-group and between-group time course- and ethanol exposure-related shifts in oligodendrocyte premyelinating and oligodendrocyte myelinating proteins without data compression by comparisons with far more abundant myelin/glial proteins. The scale bar in A reflects immunoreactivity measured in fluorescence light units, and the scale bars in B and C indicate the levels of immunoreactivity normalized to RPLPO. See Table 2 and Table 3 and Figure 3, Figure 4 and Figure 5 for statistical analysis results.

In addition to graphs depicting the time course-related differences between control and ethanol frontal lobe glial protein and RPLPO (control) expression (Figure 3A–D, Figure 4A–C and Figure 5A–D), bar plots were used to illustrate the overall effects of short-term (2 weeks or less) versus long-term (4, 6, or 8 weeks) ethanol exposures, i.e., the mean percentage increases or decreases in immunoreactivity (Figure 3E–H, Figure 4D–F and Figure 5E–H). Finally, the ELISA results were displayed with heatmaps (Figure 6), to more clearly depict the overall inter-group and time course shifts in glial marker expression.

The graphs corresponding to CNPase (Figure 3A), PLP (Figure 3B), PDGFRA (Figure 3C), and GalC (Figure 3D) exhibited similar trends, with generally higher levels of immunoreactivity in frontal lobe tissue from rats fed with ethanol-containing diets for 1 to 14 days. The post hoc tests demonstrated significant or statistical trend-wise differences after 1, 3, and/or 14 days of ethanol exposure. Day 7 uniquely showed no inter-group differences in pre-myelinating protein expression. An additional observation was that from 1 to 14 days of liquid diet feeding; pre-myelinating protein expression declined in both groups, but more steeply in controls. For the long-term cluster (4–8 weeks exposure), the mean levels of pre-myelinating proteins tended to be higher than in the 1 to 14-day cluster, and ethanol feeding resulted in significant or trend-wise reductions in PLP, PDGFRA, and GalC at the 4-week and/or 8-week time points. The time-dependent exposure effects of ethanol are shown with the bar plots (Figure 3E–H), which depict nearly global increases in pre-myelinating protein expression after short-term (1–14 days) ethanol feeding and suppression/inhibition of the same proteins in the long-term chronic ethanol-fed groups. Single-sample *t*-tests demonstrated significant short-term exposure-related increases and long-term exposure-related reductions in immunoreactivity (Table 3) and (Figure 3E–H).

The short-term phase of the experiment had progressive exposure duration-related declines in MAG1 (Figure 4A), MOG (Figure 4B), and MBP (Figure 4C), with significantly higher levels of immunoreactivity in the ethanol group at the Day 1 and Day 14 time points, and a trend reduction in MOG at the Day 3 time point. Regarding the long-term phase, the levels of myelin glycoprotein expression were elevated relative to most time points in the short-term phase. At experimental endpoints Day 28 and Day 42, the control and ethanol-exposed samples mainly had similar levels of MAG1, MOG, and MBP. However, at the final endpoint (Day 56; 8 weeks), significant inhibitory effects of ethanol were observed for MAG1 and MBP, but not MOG. The corresponding bar plots (Figure 4D–F) show predominantly short-term ethanol exposure-related stimulation of MAG1, MOG, and MBP, except for minimal responses on Day 7, whereas the long-term phase was associated with general reductions in immunoreactivity in the ethanol groups, but with significant ethanol effects limited to MOG (Table 3).

**Table 3:** Glial proteins—one-sample *T*-test.

Molecule	Early Phase Stimulation t-Statistic	*p*-Value	Late Phase Inhibition t-Statistic	*p*-Value
**CNPase**	**3.10**	**0.05**	**6.41**	**0.023**
**PLP**	**3.69**	**0.035**	*2.97*	*0.097*
**PDGFRA**	**3.72**	**0.034**	**5.56**	**0.031**
**GalC**	*2.89*	*0.063*	2.29	N.S.
**MAG1**	*2.58*	*0.082*	1.77	N.S.
**MOG**	**3.43**	**0.041**	**4.42**	**0.048**
**MBP**	*2.45*	*0.092*	1.07	N.S.
**Nestin**	1.98	N.S.	1.28	N.S.
**Vimentin**	**5.99**	**0.009**	0.85	N.S.
**GFAP**	*2.93*	*0.061*	0.71	N.S.
**RPLPO**	0.58	N.S.	0.18	N.S.

One-sample *t*-test results to assess overall stimulatory or inhibitory effects of ethanol on glial protein expression in the early (1–14 days) versus late (4–8 weeks) phases or chronic ethanol feeding. The null hypothesis was that the percentage changes in glial proteins were associated with ethanol exposure = 0. See Figure 3, Figure 4 and Figure 5. Significant differences (*p* ≤ 0.05) are highlighted with bold font. Statistical trend results (0.05 < *p* < 0.10) are italicized. N.S. = not significant.

Nestin immunoreactivity progressively increased from Day 1 to Day 28, leveled off, then declined slightly from endpoint Day 42 to Day 56. For the most part, ethanol’s effects on Nestin immunoreactivity were modest and limited to an isolated statistical trend-wise increase after Day 1, and a trend-wise reduction on Day 56 (Figure 5A). Frontal lobe vimentin immunoreactivity also progressively increased over time (Figure 5B). Statistical trend-wise increases in vimentin expression were observed in samples from ethanol-fed rats harvested on Days 1 and 14. However, by Day 56, chronic ethanol feeding led to a significant reduction in frontal lobe vimentin. GFAP immunoreactivity was similar in the control and ethanol groups during the short-term exposure phase of the study. On Day 28, the mean levels of GFAP were sharply increased in both groups and peaked in the control samples. Thereafter, control frontal lobe GFAP progressively declined. Ethanol exposures significantly muted the Day 28 peak responses and resulted in exposure duration declines in GFAP, but with similar mean levels of GFAP at endpoint Days 42 and 56 (Figure 5C). In contrast to the glial markers, RPLPO immunoreactivity was similar in control and ethanol-exposed samples, and time course-related shifts were not detected (Figure 5D). The corresponding bar plots, together with single sample *t*-tests, demonstrated significant or trend-wise ethanol-associated short-phase increases in Vimentin (Figure 5F) and GFAP (Figure 5G), but not Nestin (Figure 5E) or RPLPO (Figure 5H), and no significant overall long-term effects (Table 3).

The heatmap generated with the RPLPO ELISA data shows modest between-group variability but no distinct time course trends (Figure 6A), reflecting its suitability as a normalizing control molecule for the duplex ELISAs. The heatmap representing the aggregate specific protein data (Figure 6B) shows that CNPase, PLP, and GFAP were the most abundantly expressed glial markers in the frontal lobe. Vimentin and Nestin were initially expressed at low levels in the early/short-term phase, but during the long-term phase of the study, the levels were relatively higher. Inter-group differences were marked by variably higher ethanol-associated levels of these molecules in the early phase and lower levels in the later phase. Since PDGFRA, GALC, MAG, MOG, and MBP were expressed at relatively low levels, their side-by-side direct comparisons with glial molecules expressed at ten-fold or higher levels compressed visualization of the inter-group and time-course differences in mature myelin oligodendrocyte glycoprotein expression. A sub-heatmap that included results related to PDGFRA, GALC, MAG, MOG, and MBP was generated (Figure 6C). The most notable observation is the somewhat V-shaped time course curves marked by relatively higher protein expression levels in the very early and later time points and the lowest levels at approximately the 14-day point. In addition, there was a trend toward higher expression levels in the ethanol group in the early phase, and a reversal of that trend was observed with lower levels of protein expression in the later phase of chronic ethanol exposure.

**Figure 7:**
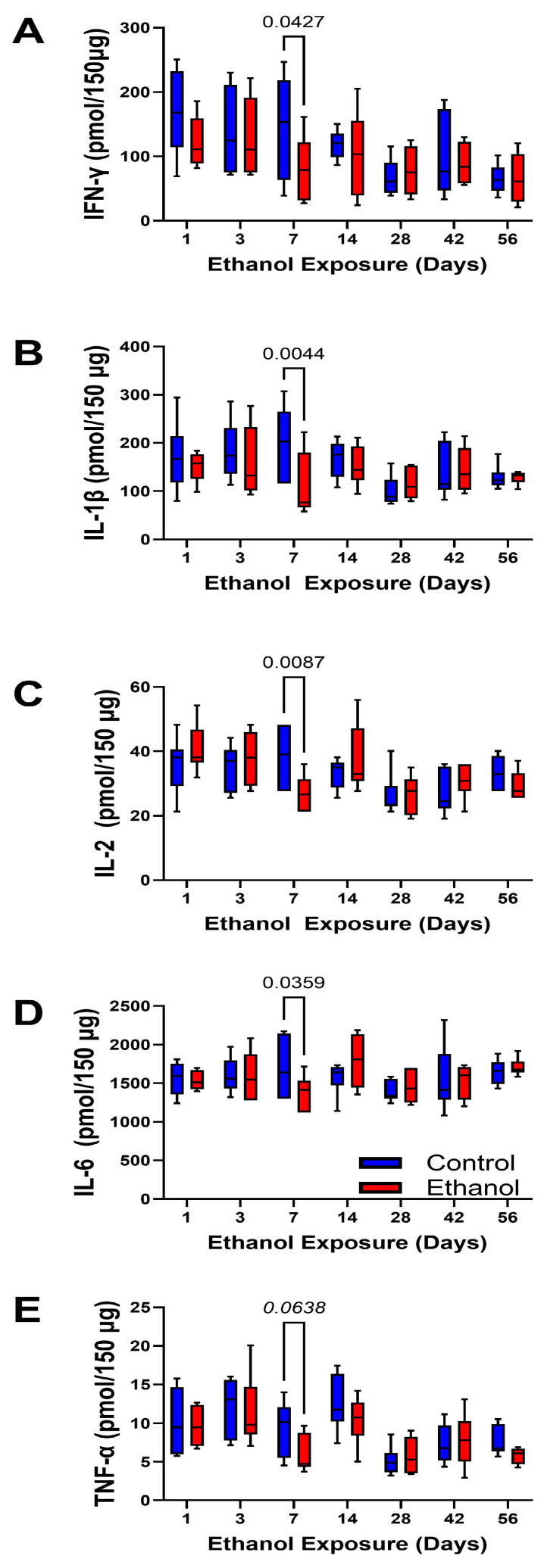
Progressive ethanol exposure effects on pro-inflammatory mediators. Frontal lobe expression of (**A**) IFN-γ, (**B**) IL-1β, (**C**) IL-2, (**D**) IL-6, and (**E**) TNF-α in adult Long Evans control (blue) and ethanol-exposed (red) rats (n = 8/group). A 5-Plex cytokine and chemokine panel measured immunoreactivity in protein samples. Graphs depict the time course shifts and corresponding differential ethanol effects (n = 8/group). Data were analyzed by two-way ANOVA (Table 4 and Table 5). Significant (*p* < 0.05) and statistical trend-wise (italics; 0.10 < *p* < 0.05) post hoc test results are displayed in the panels.

Cytokine Markers: A commercial 5-plex magnetic bead-based cytokine ELISA measured the pro-inflammatory cytokines, IFN-γ, IL-1β, IL-2, IL-6, and TNF-α in frontal lobe tissue. Two-way ANOVA tests demonstrated significant exposure duration effects on IFN-γ, IL-2, and TNF-α, and a statistical trend effect on IL-1β (Table 4). There were no significant or trend-wise effects of ethanol or ethanol × exposure duration interactions concerning any of the cytokines tested. Linear regression analysis demonstrated consistently negative slopes, reflecting progressive reductions in cytokine expression over the time course of the study (Table 5). Although the R^2^ values were generally low, the test of a non-zero slope was significant for IFN-γ, IL-2, and TNF-α in both control and ethanol samples and for IL-1β in controls (Table 5); however, we detected no significant control versus ethanol group differences in the slopes, indicating that time course-dependent declines in proinflammatory cytokine expression were similar. The graphs corresponding to the time course-dependent shifts in pro-inflammatory cytokine expression show extensive overlap between the groups (Figure 7A–E). The only significant inter-group differences by post hoc tests were detected at the 7-day time point such that the mean levels of IFN-γ (Figure 7A), IL-1β (Figure 7B), IL-2 (Figure 7C), and IL-6 (Figure 7D) were significantly reduced by ethanol, and for TNF-α, the mean level was trend-reduced by ethanol (Figure 7E).

**Table 4:** Cytokines—two-way ANOVA tests.

Molecule	Ethanol F Ratio	*p*-Value	Duration F Ratio	*p*-Value	Ethanol × Duration F Ratio	*p*-Value
**IFN-γ**	2.62	N.S.	**3.355**	**0.0062**	0.672	N.S.
**IL-1** **β**	2.498	N.S.	*2.068*	*0.068*	1.287	N.S.
**IL-2**	0.001	N.S.	**4.056**	**0.0015**	*1.957*	*0.084*
**IL-6**	0.007	N.S.	1.340	N.S.	1.187	N.S.
**TNF-α**	2.448	N.S.	**7.205**	**<0.0001**	0.660	N.S.

Frontal lobe cytokine ELISAS results analyzed by two-way ANOVA to assess effects of ethanol exposure, duration of exposure, and ethanol × duration interactive effects. Rats were maintained on 0% or 36% caloric ethanol-containing liquid diets from 1 day to 8 weeks (n = 8/time point/group). Significant differences (*p* ≤ 0.05) are highlighted with bold font. Statistical trend wise effects (0.05 < *p* < 0.10) are italicized. N.S. = not significant. Corresponding graphs with post hoc test results are displayed in Figure 7.

**Table 5:** Simple linear regression cytokine curve fitting.

Sample Group Cytokine	Equation	R^2^	F-Slope = Non-Zero	*p*-Value	Control vs. Ethanol
Control-IFN-γ	Y = −1.621 × X + 149.9	0.254	12.92	0.0009	
Ethanol-IFN-γ	Y = −0.8788 × X + 114.1	0.113	4.44	0.0423	N.S.
Control-IL-1β	Y = −1.187 × X + 181.4	0.153	7.204	0.01	
Ethanol-IL-1β	Y = −0.2625 × X + 144.0	0.012	0.481	N.S.	N.S.
Control-IL-2	Y = −0.1254 × X + 35.45	0.097	4.290	0.045	
Ethanol-IL-2	Y = −0.1525 × X + 36.19	0.123	5.480	0.024	N.S.
Control-IL-6	Y = −0.4226 × X + 1584	0.000	0.037	N.S.	
Ethanol-IL-6	Y = 1.815 × X + 1534	0.020	0.80	N.S.	N.S.
Control-TNF-α	Y = −0.08180 × X + 10.95	0.175	8.503	0.0058	
Ethanol-TNF-α	Y = −0.06645 × X + 9.602	0.140	6.331	0.016	N.S.

Simple linear regression, best fit calculations were used to test if the slopes were significantly non-zero. Significant *p*-values for non-zero slopes correspond to progressive time/exposure duration effects (reductions; negative slopes). Slopes = 0 indicate no effect of exposure duration. None of the between-group slopes were statistically significant (far right column). N.S. is not significant.

Progressive Chronic Ethanol Exposures on Insulin/IGF-1 Signaling Through Akt-mTOR: These studies characterized progressive shifts in chronic ethanol exposure effects on the expression of critical molecules, their functional phosphorylated forms, and their relative levels of phosphorylation in frontal lobe tissue. The approach employed magnetic bead-based 11-plex ELISAs to measure total and phosphorylated insulin/IGF-1-Akt-mTOR pathway signaling proteins. In addition, we measured Rictor, ^pS1591^-Rictor, Raptor, and ^pS792^-Raptor expression with duplex ELISAs. Corresponding with earlier findings in Long Evan ethanol exposure models, we did not detect significant sex differences in the signaling protein or phosphoprotein expression [29]. Therefore, data from the males and females were combined to assess the effects of ethanol, exposure duration, and ethanol × exposure duration interactions on mTOR pathway signaling molecules.

**Figure 8:**
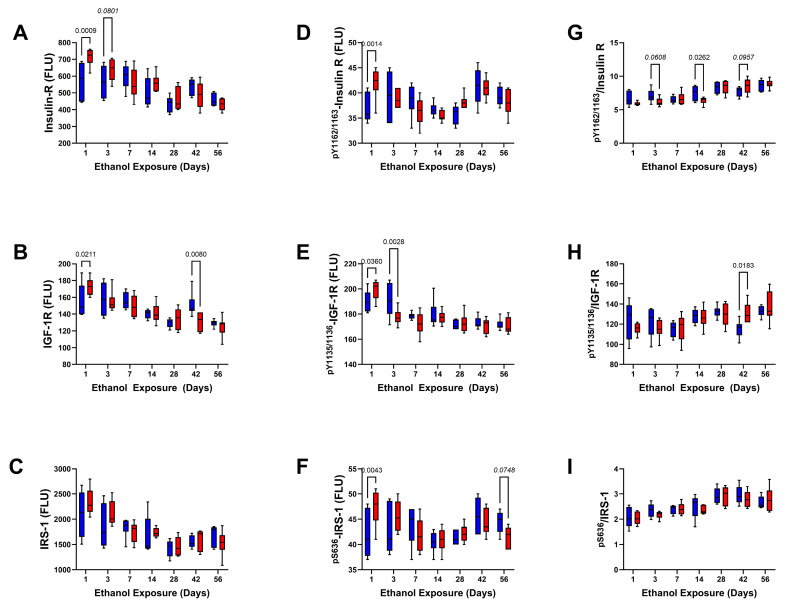
Upstream components of the Insulin/IGF-1-Akt-mTOR pathway. Commercial 11-plex magnetic bead-based ELISAs measured frontal lobe levels of (**A**) Insulin receptor (R), (**B**) IGF-1R, (**C**) IRS-1, (**D**) ^pYpY1162/1163^-Insulin-R, (**E**) ^pYpY1135/1136^-IGF-1R, and (**F**) ^pS636^-IRS-1 and the calculated relative levels of (**G**) Insulin-R, (**H**) IGF-1R, and (**I**) IRS-1 phosphorylation in adult Long Evans rats (n = 8/group) maintained for 1 to 56 days on isocaloric liquid diets containing 0% (control) or 36% ethanol. Values correspond to arbitrary fluorescent light units (FLU). Inter-group comparisons were made by a two-way ANOVA (Table 6, Table 7 and Table 8) with post hoc Tukey tests. Software generated *p*-values corresponding to significant (*p* ≤ 0.05) or statistical trend-wise (italicized; 0.10 < *p* < 0.05) differences are shown in the panels. Control-blue, Ethanol exposed-red.

Upstream Signaling Mediators: There were no significant effects of chronic ethanol feeding on the frontal lobe levels of IGF-1 receptor, IRS-1, ^pYpY1162/1163^-Insulin R, ^pYpY1135/1136^-IGF-1R, ^pS636^-IRS-1, or the relative levels of Insulin R, IGF-1R, and IRS-1 phosphorylation, but there was a statistical trend effect of ethanol on insulin receptor expression (Table 6 and Table 7). In contrast, ethanol exposure duration significantly impacted all indices, and ethanol × exposure duration interactive effects were significant for all except IRS-1. The graphs corresponding to Insulin-R (Figure 8A), IGF-1R (Figure 8B), and IRS-1 (Figure 8C) all show similarly progressive declines in immunoreactivity in the control and ethanol groups. However, post hoc tests demonstrated significant ethanol effects marked by higher levels of Insulin-R on Day 1 and Day 3, higher levels of IGF-1R on Day 1, and reduced IGF-1R on Day 42. Regarding the phosphoproteins, Day 1 of ethanol feeding significantly increased ^pYpY1162/1163^-Insulin R (Figure 8D), ^pYpY1135/1136^-IGF-1R (Figure 8E), and ^pS636^-IRS-1 (Figure 8F), but by Day 3, ethanol significantly reduced ^pYpY1135/1136^-IGF-1R. In addition, on Day 56, we observed an ethanol-associated trend-wise reduction in ^pS636^-IRS-1. The calculated relative mean levels of Insulin R tyrosine phosphorylation were reduced after 3 (trendwise) and 14 (significant) days of ethanol exposure but increased trendwise on Day 42 (Figure 8G). Ethanol also increased the mean relative level of IGF-1R tyrosine phosphorylation on Day 42 (Figure 8H). Regarding IRS-1, ethanol had no significant or trendwise effect on the relative levels of Serine phosphorylation (Figure 8I).

**Figure 9:**
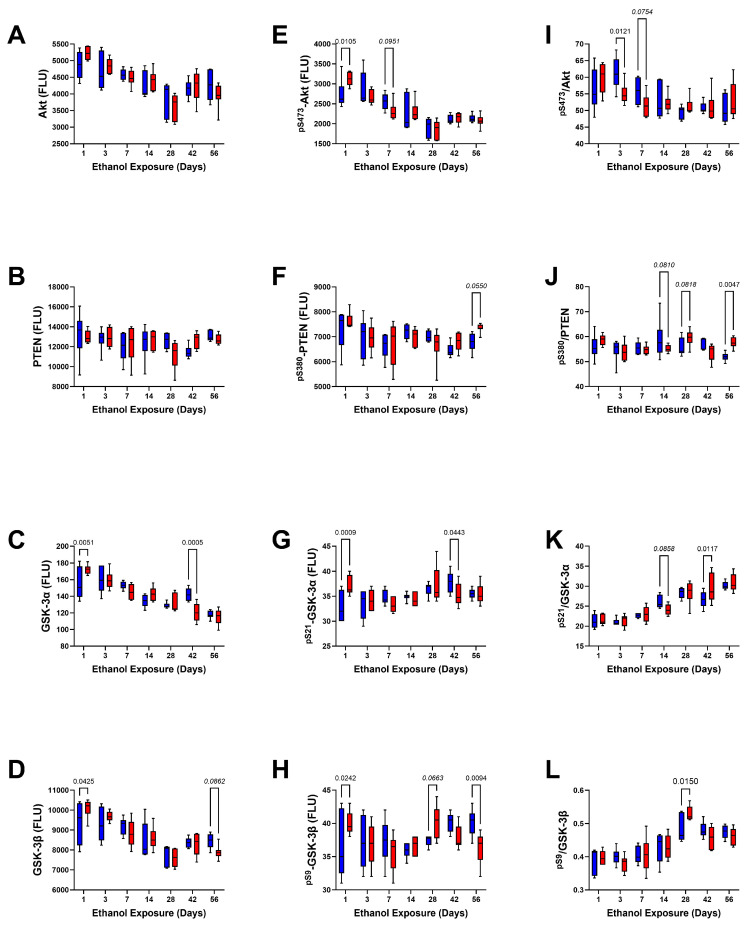
Mid-level components of the Insulin/IGF-1-Akt-mTOR pathway. Magnetic bead-based protein and phosphoprotein 11-plex ELISAs measured frontal lobe levels of (**A**) Akt, (**B**) PTEN, (**C**) GSK-3α, (**D**) GSK-3β, (**E**) ^pS473^-Akt, (**F**) ^pS380^-PTEN, (**G**) ^pS21^-GSK-3α, (**H**) ^pS9^-GSK-3β, and the calculated levels of (**I**) Akt, (**J**) PTEN, (**K**) GSK-3α, and (**L**) GSK-3β relative phosphorylation (compared with total protein) in Long Evans rats (n = 8/group) maintained for 1 to 56 days on isocaloric liquid diets containing 0% (control) or 36% ethanol. Values correspond to arbitrary fluorescent light units (FLU). Inter-group comparisons were made by a two-way ANOVA (Table 6, Table 7 and Table 8) with post hoc Tukey tests. Software calculated *p*-values represent significant (*p* ≤ 0.05) or statistical trend-wise (italicized; 0.10 < *p* < 0.05) differences are shown in the panels. Blue = Control; Red = Ethanol exposed.

Mid-Level Signaling Mediators: Akt, PTEN, GSK-3α, GSK-3β, ^pS473^-Akt, ^pS380^-PTEN, ^pS21^-GSK-3α, and ^pS9^-GSK-3β were included in this cluster (Table 6, Table 7 and Table 8 and Figure 9). There were no significant or trend-wise effects of ethanol on these proteins or phosphoproteins by two-way ANOVA. In contrast, exposure duration significantly modulated the expression of all proteins and phosphoproteins in this cluster, except for PTEN. Significant ethanol × exposure duration interactive effects were observed for GSK-3α, ^pS21^-GSK-3α, and ^pS9^-GSK-3β and a trend-wise effect was detected for ^pS473^-Akt. There were no significant or trend-wise ethanol × exposure duration interactive effects on Akt, PTEN, ^pS380^-PTEN, or GSK-3β.

**Table 6:** Akt-mTOR protein pathway—two-way ANOVA tests.

Molecule	Ethanol F Ratio	*p*-Value	Duration F Ratio	*p*-Value	Ethanol × Duration F Ratio	*p*-Value
**Insulin R**	*2.817*	*0.097*	**13.95**	**<0.0001**	**3.018**	**0.011**
**IGF-1R**	0.297	N.S.	**14.3**	**<0.0001**	**2.410**	**0.035**
**IRS-1**	0.943	N.S.	**11.89**	**<0.0001**	1.030	N.S.
**Akt**	1.332	N.S.	**16.43**	**<0.0001**	0.034	N.S.
**PTEN**	0.002	N.S.	1.789	N.S.	1.245	N.S.
**GSK-3α**	0.101	N.S.	**35.87**	**<0.0001**	**4.334**	**0.0009**
**GSK-3β**	0.005	N.S.	**18.01**	**<0.0001**	1.708	N.S.
**RPS6**	0.974	N.S.	**15.41**	**<0.0001**	**2.645**	**0.022**
**P70S6K**	1.748	N.S.	**6.583**	**<0.0001**	1.748	N.S.
**mTOR**	*2.757*	*0.10*	**52.77**	**<0.0001**	**3.002**	**0.011**
**TSC2**	0.028	N.S.	**14.83**	**<0.0001**	1.274	N.S.
**Rictor**	**19.73**	**<0.0001**	**63.21**	**<0.0001**	**6.865**	**<0.0001**
**Raptor**	**38.70**	**<0.0001**	**87.63**	**<0.0001**	**6.646**	**<0.0001**

**Figure 10:**
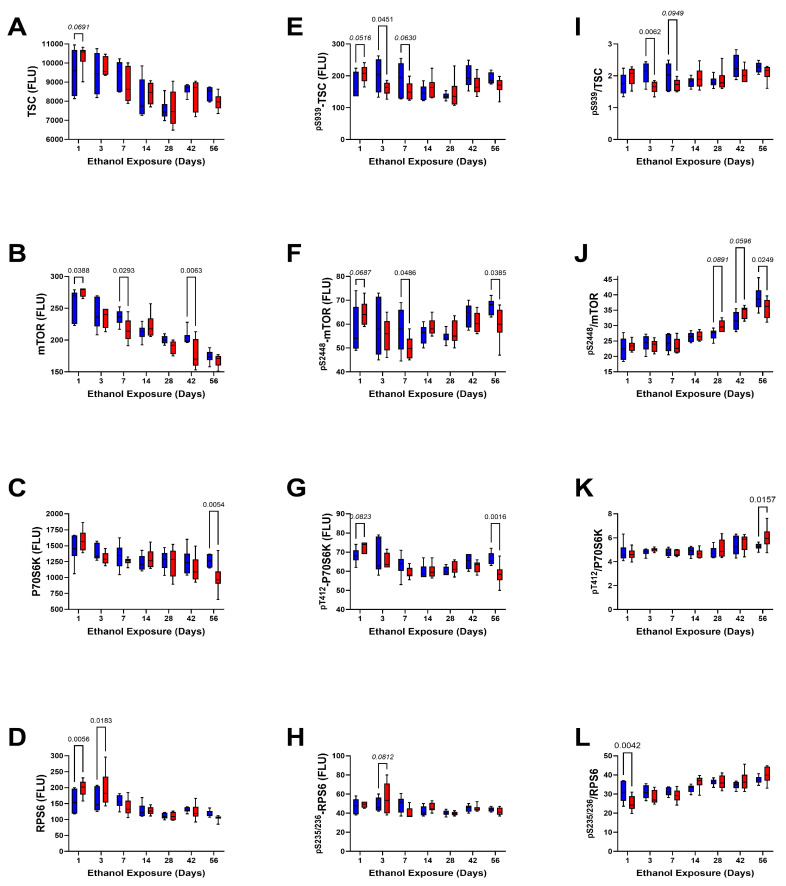
Downstream signaling through mTOR. Magnetic bead-based protein and phosphoprotein 11-plex ELISAs measured immunoreactivity to (**A**) TSC2, (**B**) mTOR, (**C**) P70S6K, (**D**) RPS6, (**E**) ^pS939^-TSC2, (**F**) ^pS2448^-mTOR, (**G**) ^pT412^-P70S6K, and (**H**) ^pS235/236^-RPS6, and the calculated relative levels of (**I**) TSC, (**J**) mTOR, (**K**) P70S6K, and (**L**) RPS6 in frontal lobe tissue (n = 8/group) from adult Long Evans rats maintained on isocaloric liquid diets containing 0% (control) or 36% ethanol for 1 to 56 days. Values correspond to arbitrary fluorescent light units (FLU). Inter-group comparisons were made by a two-way ANOVA (Table 6, Table 7 and Table 8). Software calculated *p*-values reflecting significant (*p* ≤ 0.05) or trend-wise (italicized; 0.10 < *p* < 0.05) differences are shown in the panels. Blue = Control; Red = Ethanol-exposed.

**Figure 11:**
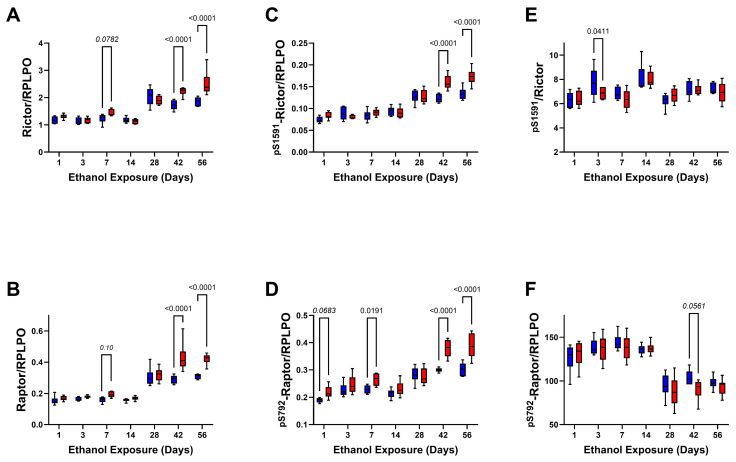
Rictor/Raptor adaptor molecules. Duplex ELISAs measured frontal lobe levels of (**A**) Rictor, (**B**) Raptor, (**C**) ^pS1591^-Rictor, and (**D**) ^pS792-^Raptor with results normalized to RPLPO. In addition, the calculated relative levels of phosphorylated (**E**) Rictor and (**F**) Raptor are shown. Inter-group comparisons (n = 8/group) were made by two-way ANOVA (Table 6, Table 7 and Table 8) and post hoc Tukey tests. Software-calculated *p*-values corresponding to significant (*p* ≤ 0.05) or statistical trend-wise (italicized; 0.10 < *p* < 0.05) differences are shown in the panels. Blue = Control; Red = Ethanol-exposed.

Two-way ANOVA test results reflecting ethanol exposure, time course/duration, and exposure × duration interactive effects on frontal lobe expression of signaling molecule proteins in the Akt/mTOR pathway. Bold font highlights significant results (*p* < 0.05). Italicized font corresponds to a statistical trend (0.05 < *p* < 0.10). N.S. = not significant. n = 8 rats/group. See Figure 8, Figure 9, Figure 10 and Figure 11 for corresponding graphs and post hoc test results. Abbreviations: R = receptor; IGF = insulin-like growth factor; IRS = insulin receptor substrate; PTEN = phosphatase and tensin homolog; GSK = glycogen synthase kinase; RPS6 = Ribosomal Protein S6; P70S6K = 70-kDa ribosomal protein S6 kinase; mTOR = mechanistic target of rapamycin; TSC = tuberous sclerosis complex 2.

**Table 7:** Akt-mTOR phospho-protein pathway—two-way ANOVA tests.

Molecule	Ethanol F Ratio	*p*-Value	Duration F Ratio	*p*-Value	Ethanol × Duration F Ratio	*p*-Value
**^pYpY1162/1163^-Insulin R**	0.219	N.S.	**5.101**	**0.0002**	**2.886**	**0.014**
**^pYpY1162/1163^-IGF-1R**	1.558	N.S.	**16.14**	**<0.0001**	**2.551**	**0.027**
**^pS636^-IRS-1**	0.405	N.S.	**3.012**	**0.011**	**2.689**	**0.021**
**^pS473^-Akt**	0.06	N.S.	**26.58**	**<0.0001**	*2.014*	*0.074*
**^pS380^-PTEN**	0.812	N.S.	**3.568**	**0.0038**	1.206	N.S.
**^pS21^-GSK-3α**	0.114	N.S.	**3.807**	**0.0024**	**3.100**	**0.009**
**^pS9^-GSK-3β**	0.068	N.S.	**2.179**	**0.05**	**3.266**	**0.0068**
**^pS235/S236^-RPS6**	0.005	N.S.	**4.122**	**0.0013**	1.749	N.S.
**^pT412^-P70S6K**	2.381	N.S.	**8.247**	**<0.0001**	**2.619**	**0.024**
**^pS2448^-mTOR**	0.952	N.S.	**4.196**	**0.001**	**2.21**	**0.05**
**^pS939^-TSC2**	1.668	N.S.	**3.168**	**0.0082**	**2.437**	**0.033**
**^pS1591^-Rictor**	**18.08**	**<0.0001**	**74.80**	**<0.0001**	**7.23**	**<0.0001**
**^pS792^-Raptor**	**42.46**	**<0.0001**	**55.49**	**<0.0001**	**5.174**	**0.0002**

Two-way ANOVA test results reflecting ethanol exposure, time course/duration, and exposure × duration interactive effects on frontal lobe expression of signaling molecule phosphoproteins in the Akt/mTOR pathway. Bold font highlights significant results (*p* < 0.05). Italicized font corresponds to a statistical trend (0.05 < *p* < 0.10). N.S. = not significant. n = 8 rats/group. See Figure 8, Figure 9, Figure 10 and Figure 11 for corresponding graphs and post hoc test results. pY = phospho-tyrosine; pS = phospho-serine; pT = phospho-threonine. See Figure 8, Figure 9, Figure 10 and Figure 11.

**Table 8:** Akt-mTOR relative phospho-protein immunoreactivity—two-way ANOVA tests.

Molecule	Ethanol F Ratio	*p*-Value	Duration F Ratio	*p*-Value	Ethanol × Duration F Ratio	*p*-Value
**^pYpY1162/1163^-/Insulin R**	1.286	N.S.	**17.91**	**0.0001**	*2.180*	*0.055*
**^pYpY1162/1163^-/IGF-1R**	0.002	N.S.	**4.880**	**0.0003**	1.578	N.S.
**^pS636^-/IRS-1**	0.638	N.S.	**12.58**	**0.0001**	0.404	N.S.
**^pS473^-/Akt**	0.258	N.S.	**7.443**	**<0.0001**	**2.263**	**0.047**
**^pS380^-/PTEN**	0.671	N.S.	1.744	N.S.	**2.913**	**0.013**
**^pS21^-/GSK-3α**	1.011	N.S.	**47.37**	**<0.0001**	1.591	N.S.
**^pS9^-/GSK-3β**	0.004	N.S.	**22.77**	**<0.0001**	1.617	N.S.
**^pS235/S236^-/RPS6**	0.285	N.S.	**16.64**	**<0.0001**	**2.845**	**0.015**
**^pT412^-/P70S6K**	1.809	N.S.	**5.680**	**<0.0001**	1.148	N.S.
**^pS2448^-/mTOR**	0.183	N.S.	**56.38**	**<0.0001**	*2.139*	*0.059*
**^pS939^-/TSC2**	*3.099*	*0.083*	**3.820**	**0.0023**	**2.451**	**0.033**
**^pS1591^-/Rictor**	1.176	N.S.	**7.679**	**<0.0001**	0.992	N.S.
**^pS792^-/Raptor**	2.187	N.S.	**43.12**	**<0.0001**	0.740	N.S.

Two-way ANOVA test results reflecting ethanol exposure, time course/duration, and exposure × duration interactive effects on frontal lobe calculated relative levels of signaling molecule phosphorylation in the Akt/mTOR pathway. Bold font highlights significant results (*p* < 0.05). Italicized font corresponds to a statistical trend (0.05 < *p* < 0.10). N.S. = not significant. n = 8 rats/group. See Figure 8, Figure 9, Figure 10 and Figure 11 for corresponding graphs and post hoc test results. pY = phospho-tyrosine; pS = phospho-serine; pT = phospho-threonine.

Graphs depicting the comparative effects of ethanol on Akt, PTEN, GSK-3α, GSK-3β, ^pS473^-Akt, ^pS380^-PTEN, ^pS21^-GSK-3α, and ^pS9^-GSK-3β over the 8-week period of investigation are shown in Figure 9. Progressive declines in Akt, GSK-3α, and GSK-3β occurred from Day 1 to Day 28, after which the levels of Akt and GSK-3β increased relative to the nadirs detected on Day 28, whereas GSK-3α declined further over time (Figure 9A,C,D). In contrast, PTEN expression remained relatively stable over the time course of the study (Figure 9B). Significant inter-group differences were marked by ethanol-associated higher levels of GSK-3α (Figure 9C) and GSK-3β (Figure 9D) at the Day 1 time point and significantly lower levels of GSK-3α at the Day 42 endpoint. An ethanol-associated trend-wise reduction in GSK-3β was observed at the Day 56 endpoint (Figure 9D).

The levels of ^pS473^-Akt declined progressively over the time course of the experiment and in concert with reductions in total Akt protein (Figure 9E). However, relative to control, ethanol significantly increased ^pS473^-Akt on Day 1 but trend-wise reduced it on Day 7. Regarding ^pS21^-GSK-3α, and ^pS9^-GSK-3β, the within-group levels varied broadly over the time course such that no overt trends were observed relative to the duration of the experiment (Figure 9G,H). However, like ^pS473^-Akt, ethanol significantly increased ^pS21^-GSK-3α and ^pS9^-GSK-3β on Day 1 and trend-wise increased ^pS9^-GSK-3β on Day 7 but significantly reduced ^pS21^-GSK-3α and ^pS9^-GSK-3β on Day 42 and Day 56, respectively. Although ^pS380^-PTEN expression remained relatively stable over the time frame of the study; on Day 56, chronic ethanol exposure significantly reduced its level relative to control (Figure 9F).

Ethanol significantly reduced the relative levels of Akt phosphorylation after 3 and 7 days exposure (Figure 9G) and trendwise reduced the relative levels of PTEN (Figure 9H) and GSK-3α (Figure 9J) phosphorylation on Day 14. In contrast, ethanol increased the relative levels of PTEN phosphorylation on Days 28 (trendwise) and 56 (significant), GSK-3α on Day 42 (significant), and GSK-3β (significant) on Day 28 (Figure 9L).

Downstream Signaling through TSC, mTOR, P70S6k, and RPS6 (Table 6, Table 7 and Table 8, Figure 10): Two-way ANOVA tests demonstrated no significant ethanol effects on TSC, P70S6K, or RPS6 but did show a trend-wise effect on mTOR. In contrast, highly significant effects of exposure duration were observed for all four molecules, and ethanol × exposure duration interactive effects were detected for mTOR and RPS6 (*p* < 0.0001) (Table 6). Regarding the phosphoproteins, two-way ANOVA tests demonstrated significant exposure duration effects on all four molecules and significant ethanol × exposure duration interactive effects on ^pS939^-TSC2, ^pS2448^-mTOR, and ^pT412^-P70S6K (Table 7). In contrast, no significant ethanol effects on phosphoprotein expression were detected. Ethanol had a trendwise effect on the relative mean level of mTOR phosphorylation, whereas exposure duration significantly impacted the relative phosphorylation of all four molecules (Table 8). Statistical trendwise or significant ethanol × exposure duration effects were observed for relative phosphorylation of RPS6 and mTOR, respectively.

The mean levels of TSC2 (Figure 10A) and mTOR (Figure 10B) progressively declined over the time course of the study. On Day 1, the mean levels of TSC1 and mTOR were elevated in the ethanol group, but on Day 7 and Day 42, mTOR was significantly reduced by ethanol. ^pS939^-TSC2 and ^pS2448^-mTOR expression varied but without conspicuous exposure-duration trends. However, ethanol-associated statistical trend-wise increases in both phosphoproteins were detected on Day 1, and significant or trend-wise reductions were observed on Days 3 (^pS939^-TSC2 and ^pS939^-/TSC2), Day 7 (^pS939^-TSC2, ^pS939^-/TSC2, and ^pS2448^-mTOR), and/or Day 56 (^pS2448^-mTOR and ^pS2448^-/mTOR) (Figure 10E,F,I,J).

RPS6 expression was significantly elevated by ethanol on Day 1 and Day 3, but thereafter, the levels remained relatively stable with no significant inter-group differences (Figure 10D). Similarly, ^pS235/236^-RPS6 immunoreactivity remained steady over the time course of the study, except for an ethanol-associated trend-wise increase on Day 3 (Figure 10H) and a reduction in the relative level of phosphorylation on Day 3 (Figure 10L). P70S6k exhibited a modest decline in immunoreactivity from Day 1 through Day 56. The reductions were similar for the control and ethanol-exposed groups except on Day 56 (8 weeks), when the mean levels of P70S6K were significantly lower in the ethanol-exposed group. The levels of ^pT412^-P70S6K were higher on Day 1 and Day 3 relative to the other time points (Figure 10C). The main effects of ethanol were to trend-wise increase in ^pT412^-P70S6K on Day 1 and significantly reduce its expression on Day 56 (Figure 10G), corresponding with the decline in P70S6K protein. However, despite reductions in both P70S6K and ^pT412^-P70S6K, the calculated relative level of P70S6K was significantly higher than control on Day 56 (Figure 10K).

Rictor and Raptor (Table 6, Table 7 and Table 8 and Figure 11). Two-way ANOVA tests demonstrated significant ethanol, exposure duration, and ethanol × exposure duration interactive effects on Rictor, ^pS1591^- Rictor, Raptor, and ^pS792^-Raptor (Table 6 and Table 7) and significant exposure duration effects on the relative levels of phosphorylation (Table 8). The progressive time course shifts in total protein and phosphoprotein expression differed distinctly from the other signaling molecules in that within the initial 14 days of the study, the levels of each molecule remained relatively stable, whereas beyond the 2-week exposure period, the levels of immunoreactivity increased (Figure 11A–D). Ethanol significantly increased Rictor, ^pS1591^- Rictor, Raptor, and ^pS792^-Raptor on Day 42 and Day 56. In contrast, the calculated relative levels of Rictor and Raptor phosphorylation were mainly similar for the control and ethanol-exposed groups (Figure 11E,F). The most notable response detected was the sharp reduction in relative phosphorylation of Raptor in the long term (28 days and beyond) compared with the earlier phase of the model.

## 4. Discussion

The main goal of this study was to examine the progressive adverse effects of chronic heavy ethanol exposures on white matter-related pathologies in ARBD over a period spanning from 1 day to 8 weeks. The working hypothesis was that the shift from early-stage, likely reversible, to late-stage ARBD is mediated by alterations in molecular signaling that mark impairments in glial functions needed to maintain mature white matter myelin. The delineation of early-stage from late-stage was based on prior evidence that in adult rats, brain atrophy based on declines in brain weight and white matter myelin staining pallor is evident after 3 weeks or more of chronic ethanol feeding [44,51]. The analytical approach included assays to characterize ethanol and exposure duration effects on myelin/oligodendrocyte protein expression, neuroinflammatory responses, and metabolic signaling through the mechanistic target of rapamycin (mTOR). The analyses focused on frontal lobe pathology and white matter degeneration due to their targeted relevance in ARBD [6,10] and contributions to neurobehavioral dysfunction and cognitive impairment [52]. ARBD severity correlates with the levels and lifetime consumption of alcohol [38,39,40], yet it can be partially reversed by cessation of alcohol exposure [41,42,43,44,53,54,55]. Apart from abstinence, few effective therapeutic measures have been identified that address oligodendrocyte/glial pathology leading to myelin loss followed by axonal damage [24] in ARBD. Knowledge Gaps persist. Filling this void could help determine if differential therapeutic interventions are required for effective management of early-stage versus late-stage ARBD.

A limitation of the present work is that it does not include neurobehavioral function assessments for correlation with the ethanol and time course-related molecular and biochemical shifts in oligodendrocyte/myelin protein expression and signaling through insulin/IGF-1/IRS-Akt-mTOR pathways. However, in many previous studies, we determined that chronic (4 weeks or longer) ethanol exposure via a liquid diet leads to significant neurobehavioral impairments on the Morris Water Maze, novel object recognition, and open field tests [20,46,56,57]. Furthermore, apart from impairments in cognitive domains, chronic ethanol exposure-mediated alterations in oligodendrocye lineage and function can lead to aberrantly increased drinking behavior associated with enhanced rather than diminished myelination in mesiotemporal structures such as the nucleus accumbens [58]. In light of the findings in this study, future studies will compare ethanol’s short-term and long-term exposure effects on neurobehavior and cognitive function to enhance the translational impact of the work. In previous related studies, we extensively evaluated the effects of sex as a biological variable in a chronic ethanol exposure model [29,57]. The main findings were that the females, irrespective of ethanol exposure, were smaller than males over the entire 9-week time course of the study [29,57], corresponding with other published works [59,60]. However, there were no significant effects of sex on BAC, brain weight, neurobehavioral/cognitive performance, or the expression of inflammatory, oxidative stress, or intracellular signaling molecules [29,57]. Therefore, the results obtained from male and female rats were combined for data analysis.

This study was initiated with rats that were 6 weeks old because over 90% of the adult brain weight is achieved by that age [61,62], providing an opportunity to monitor progressive alcohol-related brain atrophy. Of note is that a significant reduction in brain weight was first detected after 4 weeks of chronic ethanol feeding and persisted to the final endpoint of the study (8 weeks). This effect was not associated with time-dependent shifts in blood alcohol concentrations, which remained stable from Day 3 through Day 56, or changes in liver weight, which never differed significantly between the control and ethanol-exposed groups. Therefore, other factors must account for brain atrophy, which previous studies linked to impaired performance on neurobehavioral tests [29,56,57]. On the other hand, the atrophy linked to duration of chronic ethanol feeding is reminiscent of the human studies showing that ARBD severity correlates with cumulative lifetime consumption of alcohol [38,39,40]. By way of comparison with humans, after Day 1, the blood alcohol levels (BACs) in the ethanol-fed rats ranged from 70 mg/dL to 322 mg/dL and averaged between 115.7 and 153.2 mg/dL. In humans, impairments in memory, judgment, and balance occur with BACs between 100 and 200 mg/dL, confusion and disorientation result with BACs between 200 and 300 mg/dL, and disordered breathing, stupor, and finally coma occur with BACs above 350 mg/dL [63]. Therefore, the BAC levels were within the range expected to impair memory as reported previously using this model [20,46,56,57].

The histopathological studies of frontal lobe corpus callosum white matter revealed only modest reductions in myelin staining intensity during the initial 2 weeks but notable reductions in myelin staining intensity after 6 or 8 weeks of chronic ethanol exposure. These observations correspond with previous findings of white matter degeneration in Long Evans rat alcohol exposure models [44,64] and in humans with alcohol use disorder [10,39,65,66,67]. In humans, neuroimaging studies have shown distinct abnormalities in white matter integrity in ARBD [39,54,65,68,69,70]. In addition, biochemical analyses have linked white matter ARBD in rats and humans to alterations in myelin lipid content and composition [71,72,73,74,75]. Damage to white matter myelin has also been linked to axonal degeneration, loss of oligodendrocytes, and increased fibrillary astrocytes [6,10,14,64]. The potential role of neuroinflammation was investigated by measuring proinflammatory cytokines and chemokines in frontal lobe tissue. Contrary to previous expectations, we did not observe significant pro-inflammatory responses in the ethanol-exposed brains. Instead, at the Day 7 endpoint, we detected significant reductions in cytokine/chemokine expression, and otherwise, the inflammatory indices were similar in control and ethanol samples. Therefore, it is unlikely that neuroinflammation was the critical factor governing brain atrophy and white matter degeneration. These findings are discordant with other reports demonstrating significant neuroinflammatory responses in alcohol-exposure models [76,77]. However, notable differences in the study designs were that robust neuroinflammation was reported in younger-developing or adolescent rats models [78,79,80] and when the ethanol exposures were binge, chronic + binge, or associated with other deleterious exposures [81,82]. This suggests that factors contributing to ethanol-mediated neuroinflammation include host susceptibility related to the host maturation stage and bolus delivery of high neurotoxic doses of ethanol.

Frontal lobe white matter glial proteins exhibited distinctly patterned time course and ethanol exposure × duration interactive effects that delineated early responses within the initial two weeks from later responses after 4 weeks. For the most part, the expression levels of non-myelinating (CNPase, PLP, PDGFRA, and GalC) and myelinating (MAG1, MOG, and MBP) molecules were increased during the early or short-duration ethanol exposures but inhibited after prolonged chronic exposure. Inhibition of myelin/glial protein expression has been reported for chronic ethanol exposure models [18,55,83] and in the brains of humans with alcohol use disorder [84], but their up-regulation in the acute to subacute intervals was not previously demonstrated. Although the study was designed to study adult brains and minimize the effects of development, time course studies in rodents cannot exclude normal age-associated contributions [83,85]. Correspondingly, the declines in immunoreactivity between Day 1 and Day 14 in both the control and ethanol-exposed groups and the generally higher levels of myelin glycoprotein expression at the 4-week and beyond endpoints most likely reflect normal shifts in oligodendrocyte-myelination functions linked to adult brain maturation [83,86]. However, the paired timepoint analyses suggest that ethanol significantly altered these normal trajectories by enhancing protein expression in the acute to subacute phases and suppressing responses in later chronic phases.

Previous experiments conducted in 3-week and 8-week chronic + binge ethanol exposure models also demonstrated ethanol-related alterations in the expression of myelin -oligodendrocyte glycoprotein immunoreactivity or mRNA [55]. Superimposed binge ethanol administration resulted in more striking inhibitory shifts in myelin glycoprotein expression compared with lower moderate and chronic exposures. Importantly, ethanol’s effects were also linked to significant alterations in myelin lipid phospholipid and sphingolipid composition, which were detected within 3 weeks of exposure and further shifted by the 8-week time point [44]. As observed in the present study, ethanol exposure-duration worsened the severity of white matter atrophy and markedly reduced myelin staining [44]. Therefore, ethanol-mediated shifts in oligodendrocyte-myelin glycoprotein expression are associated with significant alterations in myelin lipid composition and white matter atrophy which occur within the timeframe of cognitive impairment. The earlier studies showed that abstinence only partly reversed the myelin lipid abnormalities [44], marking a difficult-to-reverse chronic disease state. However, in a later study, treatment with myriocin, a ceramide inhibitor, partially resolved ethanol’s inhibitory effects on sphingomyelin expression and the impairments in neurobehavioral function [56]. Although there is no immediate explanation for these observed transitions from early-phase to late-phase ethanol-associated effects on oligodendrocyte-myelin protein expression, we hypothesize that multiple signaling pathways including Notch [87] also become dysregulated, perhaps via epigenetic mediators [84,88]. Furthermore, the early activation or up-regulated oligodendrocyte-myelin glycoprotein expression likely reflects neurotoxic injury caused by acute ethanol exposure, whereas the later inhibitory effects correspond to the establishment of long-term white matter myelin pathology linked to brain atrophy as observed herein. Additional studies are needed to directly address the underlying mechanistic questions.

Astroglial (nestin, vimentin, GFAP) protein expression also exhibited two distinct trajectories corresponding to the early (initial 2 weeks) versus late (last 6 weeks) phases of the study. Nestin, vimentin, and GFAP were initially expressed at lower levels and either gradually increased (Nestin and Vimentin) or remained stable (GFAP) from Day 1 to Day 14. Subsequently, the expression levels shifted upward and either remained stable (Nestin and Vimentin) or progressively declined (GFAP). Most of the intergroup differences corresponded to trend effects, except for the significantly lower expression of Vimentin on Day 56 and GFAP on Day 28. Nonetheless, like the oligodendrocyte/myelin proteins, the early-phase component of the study was associated with relatively increased expression of Nestin, Vimentin, and GFAP in the ethanol-exposed samples, whereas in the long-term period, Vimentin and GFAP were reduced at experimental endpoints Days 28 and 56. The ethanol-mediated late-phase reductions in vimentin and GFAP are of interest because astrocytes provide structural/architectural support for ensuring blood–brain barrier integrity and mediating CNS synaptic plasticity and regeneration [89,90,91]. Alcohol exposures impair the blood–brain barrier integrity [92,93,94] and synaptic plasticity [95]. The findings herein suggest that the transition from acute/subacute to chronic phases of ARBD is mediated in part by the emergence of astroglial functional impairments. Additional studies are needed to identify the “switches” that trigger the onset of the ARBD neurodegeneration cascade.

A major goal of this study was to characterize the early- versus late-stage effects of heavy ethanol consumption on the insulin/IGF-1-Akt-mTOR pathway in relation to brain atrophy. This research direction was guided by evidence that mTOR networks support oligodendrocytes and myelination [30,33,96,97] and that they are impaired by chronic alcohol exposure [27,28,29,98]. The knowledge gap pertains to understanding the time course of progressive alterations in signaling because, for the most part, previous experiments were conducted using single-endpoint exposure models. The main effects observed with respect to the upstream and intermediate components of the pathway were that the expression levels progressively declined over the 8-week time course. Although, by 6 weeks of age, the rat brain nearly achieves its full adult size [24], its continued enlargement over the time course of this study reflects ongoing maturation, mainly due to increased white matter myelination. The very early, primarily Day 1 response to ethanol, in which the mean levels of insulin R, IGF-1R, ^pS473^-Akt, ^pS21^-GSK-3α, and ^pS9^-GSK-3β were increased, favored pro-growth and pro-survival signaling, possibly reflecting adaptive/compensatory responses to ethanol’s acute neurotoxic effects. It is also noteworthy that the shifts in phosphoprotein expression mainly accompanied concordant changes in the non-phosphorylated proteins, suggesting that ethanol’s early effects on pathway activation were largely driven by changes in protein expression rather than selective increases or reductions in phosphorylation state. This point is reinforced by the largely absent significant or trendwise effects of ethanol exposure on the levels of relative signaling molecules phosphorylation.

The notable long-term effects of ethanol, including significant reductions in IGF-1R, GSK-3α, and ^pS21^-GSK-3α on Day 42, and GSK-3β and ^pS9^-GSK-3β on Day 56, would likely have produced mixed rather than predominantly pro- and anti-metabolic/growth/survival effects. Overall, the inhibitory effects of chronic ethanol exposure on the upstream and midstream signaling mechanisms in the insulin/IGF-/IRS/Akt pathway in the adult brain were relatively modest compared with the findings in developing postnatal and adolescent brains, which exhibit prominent impairments in signaling at multiple levels within the cascade [20,46,51,99]. Those differences reinforce the concept that the immature CNS is substantially more vulnerable than the adult brain is to the neurotoxic and degenerative effects of ethanol. In addition, the findings suggest that the underlying mechanisms of ARBD differ in mature adult versus immature brains, yet CNS white matter is targeted in both. Further probing of downstream mediators was conducted to determine the mechanistic role of dysregulated mTOR signaling in relation to brain atrophy in ARBD.

Analysis of the mTOR pathway revealed ethanol exposure and time course-dependent declines in TSC, mTOR, RPS6, and P70S6k proteins, without parallel reductions in the corresponding phosphoproteins. As observed for the other signaling molecules, ethanol broadly increased the levels of these proteins and their respective phosphoproteins at endpoint Days 1 and/or 3. In contrast, the 6- and 8-week experimental endpoints were associated with reduced mTOR-P70S6K signaling, marked by significant reductions in mTOR, ^pS2448^-mTOR, P70S6K, and ^pT412^-P70S6K. Of note is that RPS6 protein and phosphoprotein were not significantly altered by ethanol. Further analysis showed that chronic heavy ethanol exposures were associated with significantly increased frontal lobe expression of Rictor, ^pS1591^-Rictor, Raptor, and ^pS792^-Raptor at the Day 42 and Day 56 experimental endpoints. These observations provide new and highly relevant information about the mechanisms of brain atrophy and white matter degeneration in ARBD. Rictor and Raptor are critical components of the mTORC1/2 complexes and serve to stimulate downstream signaling through P70S6K and RPS6 [100,101,102]. However, Serine phosphorylation of Rictor/Raptor inhibits mTORC [103]. Ethanol-mediated increases in the total and phosphorylated levels of both Rictor and Raptor, together with inhibition of mTOR and ^pS2448^-mTOR, likely impaired mTOR/mTORC signaling, as was evidenced by the sharp reductions in P70S6k and ^pT412^-P70S6K.

Given the critical roles of mTOR signaling for ongoing myelination, myelin maintenance, oligodendrocyte function [28,30,33,96,97,98], synaptic plasticity [104,105,106,107,108], and metabolism [36,109,110], the long-term chronic ethanol-associated inhibitory effects on this pathway could account for the associated broad reductions in myelin glycoprotein expression and white matter myelin staining. The finding of long-term inhibitory effects of ethanol on mTOR signaling in the brain is consistent with our previous observations in a chronic moderate-dose ethanol-exposure model in adolescent rats [29]. The consequences likely included white matter atrophy, which has been well documented following chronic ethanol exposure [8,10,44,56,67,87,111]. Intact signaling through mTOR/mTORC/P70S6K is crucial for maintaining oligodendrocyte functions required for white matter integrity, together with a broad range of cellular processes related to neuronal plasticity, RNA translation, cell survival, energy, and mitochondrial function [28,100,101,112]. Impairments of mTOR/mTORC signaling cause oligodendrocyte dysfunction, compromising myelination and myelin homeostasis [33,97,113]. In the present study, we observed significant chronic, long-term/late-phase ethanol exposure-associated inhibition of mTOR signaling with reductions in phospho-mTOR, increased Raptor and Rictor phosphorylation, and reduced activation of P70S6K, which were not present during the early phases of ethanol exposure. These findings suggest that the critical factor governing transition from acute/subacute to chronic ARBD is the establishment of sustained mTOR pathway inhibition through mTORC1, mTORC2, and P70S6K. However, the significance of the findings herein could be strengthened by mechanistic experiments that directly assess the impact of mTOR inhibition on oligodendrocyte/myelin protein and mRNA expression in the context of chronic ethanol exposure. Nonetheless, the information gained from this research together with earlier publications highlights the need to develop novel strategies to support mTOR/mTORC signaling to prevent white matter ARBD. It is also noteworthy that similar white matter abnormalities have been linked to Alzheimer’s disease [114], suggesting that mTOR/mTORC targeted therapeutic interventions may benefit other neurodegenerative diseases in which white matter degeneration is a dominant feature.

## 5. Conclusions

This study characterized the progressive nature of ARBD starting from a very early period (1 day exposure) and ending after 8 weeks of chronic high-dose ethanol feeding in adult Long Evans rats. One of the main goals was to delineate the differential effects of short-term versus long-term ethanol exposure to identify critical factors linked to sustained atrophy, white matter degeneration, and neurobehavioral dysfunction. The hope was that time course and pathophysiological factors linked to chronic, difficult-to-reverse brain atrophy would be determined, enabling guidance with respect to future monitoring, therapeutic and preventive measures. The investigations clearly delineated the effects of early and short-term versus long-term chronic effects of heavy alcohol exposure in that the early period in which brain atrophy and white matter myelin loss were minimal was associated with activation of oligodendrocyte and insulin/IGF/Akt-mTOR pathways, whereas the chronic phase, coinciding with brain atrophy and white matter degeneration, was marked by impaired signaling through mTOR. Among the major strengths of this article is that the analyses attended to progressive, multipronged brain pathologies over a broad timeframe, rather than a single time point. Although the studies were focused on one brain region, prior investigations included other structures such as the temporal lobe with hippocampus and the cerebellum. Neurobehavioral function tests were not utilized because the outcomes have already been well documented in our model, and our primary goal was to emphasize the mechanisms of chronic ARBD and identify differences between the early and late-stages of progressive disease. Future studies will examine time course-dependent effects of ethanol on myelin profiles, given the important roles membrane lipids have in maintaining the structural and functional integrity of white matter across the lifespan.

## Data Availability

The data underlying this article will be shared on reasonable request to the corresponding author.

## Supplementary Materials

The following supporting information can be downloaded at: https://www.mdpi.com/article/10.3390/biom15030413/s1, Supplementary Table S1: Critical Reagent and Instrument Sources; Supplementary Table S2: Antibody Sources and Preparations; Supplementary Table S3A: 11-Plex Akt-mTOR Pathway (Proteins and Phosphoproteins); Supplementary Table S3B: Rat Cytokine 5-Plex Antibodies; Supplementary Table S4: mTOR Signaling Proteins and Brain Functions [18,19,30,31,97,100,101,104,114,115,116,117,118,119,120,121,122,123,124,125,126,127,128,129,130,131,132,133,134,135,136,137,138,139,140,141,142,143,144,145,146,147,148,149,150,151,152,153,154,155,156,157,158,159,160,161,162,163,164,165,166,167,168,169,170,171,172,173,174,175,176,177,178,179,180,181,182,183,184,185].

## References

[1] Bouchery E.E., Harwood H.J., Sacks J.J., Simon C.J., Brewer R.D. (2011). Economic costs of excessive alcohol consumption in the U.S., 2006. Am. J. Prev. Med..

[2] Rehm J., Baliunas D., Borges G.L., Graham K., Irving H., Kehoe T., Parry C.D., Patra J., Popova S., Poznyak V. (2010). The relation between different dimensions of alcohol consumption and burden of disease: An overview. Addiction.

[3] Sacks J.J., Gonzales K.R., Bouchery E.E., Tomedi L.E., Brewer R.D. (2015). 2010 National and State Costs of Excessive Alcohol Consumption. Am. J. Prev. Med..

[4] Stahre M., Roeber J., Kanny D., Brewer R.D., Zhang X. (2014). Contribution of excessive alcohol consumption to deaths and years of potential life lost in the United States. Prev. Chronic Dis..

[5] Brust J. (2010). Ethanol and Cognition: Indirect Effects, Neurotoxicity and Neuroprotection: A Review. Int. J. Environ. Res. Public Health.

[6] de la Monte S.M., Kril J.J. (2014). Human alcohol-related neuropathology. Acta Neuropathol..

[7] Fortier C.B., Leritz E.C., Salat D.H., Lindemer E., Maksimovskiy A.L., Shepel J., Williams V., Venne J.R., Milberg W.P., McGlinchey R.E. (2014). Widespread effects of alcohol on white matter microstructure. Alcohol. Clin. Exp. Res..

[8] de la Monte S.M. (1988). Disproportionate atrophy of cerebral white matter in chronic alcoholics. Arch Neurol..

[9] Harper C., Dixon G., Sheedy D., Garrick T. (2003). Neuropathological alterations in alcoholic brains. Studies arising from the New South Wales Tissue Resource Centre. Prog. Neuropsychopharmacol. Biol. Psychiatry.

[10] Sutherland G.T., Sheedy D., Kril J.J. (2014). Neuropathology of alcoholism. Handb. Clin. Neurol..

[11] Kuhn S., Gritti L., Crooks D., Dombrowski Y. (2019). Oligodendrocytes in Development, Myelin Generation and Beyond. Cells.

[12] Simons M., Nave K.A. (2015). Oligodendrocytes: Myelination and Axonal Support. Cold Spring Harb. Perspect. Biol..

[13] Benjamins J.A., Nedelkoska L., Lisak R.P., Hannigan J.H., Sokol R.J. (2011). Cytokines reduce toxic effects of ethanol on oligodendroglia. Neurochem. Res..

[14] Creeley C.E., Dikranian K.T., Johnson S.A., Farber N.B., Olney J.W. (2013). Alcohol-induced apoptosis of oligodendrocytes in the fetal macaque brain. Acta Neuropathol. Commun..

[15] Joseph D’Ercole A., Ye P. (2008). Expanding the mind: Insulin-like growth factor I and brain development. Endocrinology.

[16] Zeger M., Popken G., Zhang J., Xuan S., Lu Q.R., Schwab M.H., Nave K.A., Rowitch D., D’Ercole A.J., Ye P. (2007). Insulin-like growth factor type 1 receptor signaling in the cells of oligodendrocyte lineage is required for normal in vivo oligodendrocyte development and myelination. Glia.

[17] Chesik D., De Keyser J., Wilczak N. (2008). Insulin-like growth factor system regulates oligodendroglial cell behavior: Therapeutic potential in CNS. J. Mol. Neurosci..

[18] Ewenczyk A., Ziplow J., Tong M., Le T., de la Monte S.M. (2012). Sustained Impairments in Brain Insulin/IGF Signaling in Adolescent Rats Subjected to Binge Alcohol Exposures during Development. J. Clin. Exp. Pathol..

[19] Le T., Tong M., Nguyen V., de la Monte S.M. (2013). PPAR Agonist Rescue of Ethanol-Impaired Brain Insulin Signaling: Cerebellar Slice Culture Model. J. Drug Alcohol Res..

[20] Tong M., Yu R., Deochand C., de la Monte S.M. (2015). Differential Contributions of Alcohol and the Nicotine-Derived Nitrosamine Ketone (NNK) to Insulin and Insulin-Like Growth Factor Resistance in the Adolescent Rat Brain. Alcohol Alcohol..

[21] Spear L. (2013). The Teenage Brain: Adolescents and Alcohol. Curr. Dir. Psychol. Sci..

[22] de Goede J., van der Mark-Reeuwijk K.G., Braun K.P., le Cessie S., Durston S., Engels R.C.M.E., Goudriaan A.E., Moons K.G.M., Vollebergh W.A.M., de Vries T.J. (2021). Alcohol and Brain Development in Adolescents and Young Adults: A Systematic Review of the Literature and Advisory Report of the Health Council of The Netherlands. Adv. Nutr..

[23] Lam V.Y.Y., Raineki C., Ellis L., Yu W., Weinberg J. (2018). Interactive effects of prenatal alcohol exposure and chronic stress in adulthood on anxiety-like behavior and central stress-related receptor mRNA expression: Sex- and time-dependent effects. Psychoneuroendocrinology.

[24] Lees B., Meredith L.R., Kirkland A.E., Bryant B.E., Squeglia L.M. (2020). Effect of alcohol use on the adolescent brain and behavior. Pharmacol. Biochem. Behav..

[25] Lees B., Mewton L., Jacobus J., Valadez E.A., Stapinski L.A., Teesson M., Tapert S.F., Squeglia L.M. (2020). Association of Prenatal Alcohol Exposure with Psychological, Behavioral, and Neurodevelopmental Outcomes in Children from the Adolescent Brain Cognitive Development Study. Am. J. Psychiatry.

[26] Pfefferbaum A., Sullivan E.V., Pohl K.M., Bischoff-Grethe A., Stoner S.A., Moore E.M., Riley E.P. (2023). Brain Volume in Fetal Alcohol Spectrum Disorders Over a 20-Year Span. JAMA Netw. Open.

[27] Li Q., Ren J. (2007). Chronic alcohol consumption alters mammalian target of rapamycin (mTOR), reduces ribosomal p70s6 kinase and p4E-BP1 levels in mouse cerebral cortex. Exp. Neurol..

[28] de la Monte S.M., Tong M., Delikkaya B. (2023). Differential Early Mechanistic Frontal Lobe Responses to Choline Chloride and Soy Isoflavones in an Experimental Model of Fetal Alcohol Spectrum Disorder. Int. J. Mol. Sci..

[29] Yalcin E.B., Tong M., Delikkaya B., Pelit W., Yang Y., de la Monte S.M. (2024). Differential effects of moderate chronic ethanol consumption on neurobehavior, white matter glial protein expression, and mTOR pathway signaling with adolescent brain maturation. Am. J. Drug Alcohol Abus..

[30] Figlia G., Gerber D., Suter U. (2018). Myelination and mTOR. Glia.

[31] Narayanan S.P., Flores A.I., Wang F., Macklin W.B. (2009). Akt signals through the mammalian target of rapamycin pathway to regulate CNS myelination. J. Neurosci..

[32] Zhou H., Huang S. (2010). The complexes of mammalian target of rapamycin. Curr Protein Pept Sci.

[33] Tyler W.A., Gangoli N., Gokina P., Kim H.A., Covey M., Levison S.W., Wood T.L. (2009). Activation of the mammalian target of rapamycin (mTOR) is essential for oligodendrocyte differentiation. J. Neurosci..

[34] Goebbels S., Oltrogge J.H., Kemper R., Heilmann I., Bormuth I., Wolfer S., Wichert S.P., Mobius W., Liu X., Lappe-Siefke C. (2010). Elevated phosphatidylinositol 3,4,5-trisphosphate in glia triggers cell-autonomous membrane wrapping and myelination. J. Neurosci..

[35] Dubois J., Dehaene-Lambertz G., Kulikova S., Poupon C., Huppi P.S., Hertz-Pannier L. (2014). The early development of brain white matter: A review of imaging studies in fetuses, newborns and infants. Neuroscience.

[36] Laplante M., Sabatini D.M. (2012). mTOR Signaling. Cold Spring Harb. Perspect. Biol..

[37] Jacobus J., Squeglia L.M., Bava S., Tapert S.F. (2013). White matter characterization of adolescent binge drinking with and without co-occurring marijuana use: A 3-year investigation. Psychiatry Res..

[38] Estruch R., Nicolas J.M., Salamero M., Aragon C., Sacanella E., Fernandez-Sola J., Urbano-Marquez A. (1997). Atrophy of the corpus callosum in chronic alcoholism. J. Neurol. Sci..

[39] Pfefferbaum A., Sullivan E.V. (2002). Microstructural but not macrostructural disruption of white matter in women with chronic alcoholism. Neuroimage.

[40] Woods A.J., Porges E.C., Bryant V.E., Seider T., Gongvatana A., Kahler C.W., de la Monte S., Monti P.M., Cohen R.A. (2016). Current Heavy Alcohol Consumption is Associated with Greater Cognitive Impairment in Older Adults. Alcohol. Clin. Exp. Res..

[41] Estilaei M.R., Matson G.B., Payne G.S., Leach M.O., Fein G., Meyerhoff D.J. (2001). Effects of abstinence from alcohol on the broad phospholipid signal in human brain: An in vivo 31P magnetic resonance spectroscopy study. Alcohol. Clin. Exp. Res..

[42] Bartsch A.J., Homola G., Biller A., Smith S.M., Weijers H.G., Wiesbeck G.A., Jenkinson M., De Stefano N., Solymosi L., Bendszus M. (2007). Manifestations of early brain recovery associated with abstinence from alcoholism. Brain A J. Neurol..

[43] Mon A., Delucchi K., Durazzo T.C., Gazdzinski S., Meyerhoff D.J. (2011). A mathematical formula for prediction of gray and white matter volume recovery in abstinent alcohol dependent individuals. Psychiatry Res..

[44] Yalcin E.B., McLean T., Tong M., de la Monte S.M. (2017). Progressive white matter atrophy with altered lipid profiles is partially reversed by short-term abstinence in an experimental model of alcohol-related neurodegeneration. Alcohol.

[45] Gazdzinski S., Durazzo T.C., Mon A., Yeh P.H., Meyerhoff D.J. (2010). Cerebral white matter recovery in abstinent alcoholics—A multimodality magnetic resonance study. Brain A J. Neurol..

[46] Tong M., Ziplow J.L., Mark P., de la Monte S.M. (2022). Dietary Soy Prevents Alcohol-Mediated Neurocognitive Dysfunction and Associated Impairments in Brain Insulin Pathway Signaling in an Adolescent Rat Model. Biomolecules.

[47] DaDalt A.A., Bonham C.A., Lotze G.P., Luiso A.A., Vacratsis P.O. (2022). Src-mediated phosphorylation of the ribosome biogenesis factor hYVH1 affects its localization, promoting partitioning to the 60S ribosomal subunit. J. Biol. Chem..

[48] Liu H.T., Zou Y.X., Zhu W.J., Sen L., Zhang G.H., Ma R.R., Guo X.Y., Gao P. (2022). lncRNA THAP7-AS1, transcriptionally activated by SP1 and post-transcriptionally stabilized by METTL3-mediated m6A modification, exerts oncogenic properties by improving CUL4B entry into the nucleus. Cell Death Differ..

[49] Xie J.J., Jiang Y.Y., Jiang Y., Li C.Q., Lim M.C., An O., Mayakonda A., Ding L.W., Long L., Sun C. (2018). Super-Enhancer-Driven Long Non-Coding RNA LINC01503, Regulated by TP63, Is Over-Expressed and Oncogenic in Squamous Cell Carcinoma. Gastroenterology.

[50] White N.M., Byrne J.H. (2008). 3.02—Multiple Memory Systems in the Brain: Cooperation and Competition. Learning and Memory: A Comprehensive Reference.

[51] Cohen A.C., Tong M., Wands J.R., De La Monte S.M. (2007). Insulin and Insulin-Like Growth Factor Resistance With Neurodegeneration in an Adult Chronic Ethanol Exposure Model. Alcohol. Clin. Exp. Res..

[52] Chanraud S., Martelli C., Delain F., Kostogianni N., Douaud G., Aubin H.J., Reynaud M., Martinot J.L. (2007). Brain morphometry and cognitive performance in detoxified alcohol-dependents with preserved psychosocial functioning. Neuropsychopharmacol. Off. Publ. Am. Coll. Neuropsychopharmacol..

[53] Onoda A., Maruki Y., Matsuzaki M., Narabayasi Y., Sawada M., Iwasaki A., Enokida M., Kanaya M., Akiyama H., Yamauchi T. (2000). Abstinence from drink ameliorated cerebral blood flow and vasoreactivity in patients with chronic alcoholism. Keio J. Med..

[54] Pfefferbaum A., Adalsteinsson E., Sullivan E.V. (2006). Dysmorphology and microstructural degradation of the corpus callosum: Interaction of age and alcoholism. Neurobiol. Aging.

[55] Yalcin E.B., Tong M., de la Monte S.M. (2018). Altered Oligodendroglial and Neuroglial Gene Expression in Adult Rat Cerebral White Matter Following Short- and Long-Term Ethanol Exposures and Abbreviated Abstinence. J. Drug Alcohol Res..

[56] Homans C., Yalcin E.B., Tong M., Gallucci G., Bautista D., Moriel N., Monte S.d.l. (2022). Therapeutic Effects of Myriocin in Experimental Alcohol-Related Neurobehavioral Dysfunction and Frontal Lobe White Matter Biochemical Pathology. J. Behav. Brain Sci..

[57] Yalcin E.B., Delikkaya B.N., Pelit W., Tong M., De La Monte S.M., Rounds S. (2022). The Differential Effects of Chronic Alcohol and Cigarette Smoke Exposures on Cognitive-Behavioral Dysfunction in Long Evans Rats. J. Behav. Brain Sci..

[58] Liran M., Fischer I., Elboim M., Rahamim N., Gordon T., Urshansky N., Assaf Y., Barak B., Barak S. (2025). Long-term excessive alcohol consumption enhances myelination in the mouse nucleus accumbens. J. Neurosci..

[59] Quiros Cognuck S., Reis W.L., Silva M., Debarba L.K., Mecawi A.S., de Paula F.J.A., Rodrigues Franci C., Elias L.L.K., Antunes-Rodrigues J. (2020). Sex differences in body composition, metabolism-related hormones, and energy homeostasis during aging in Wistar rats. Physiol. Rep..

[60] Santiago H.A., De Pierro L.R., Reis R.M., Caluz A.G., Ribeiro V.B., Volpon J.B. (2015). Allometric relationships among body mass, MUZZLE-tail length, and tibia length during the growth of Wistar rats. Acta Cir. Bras..

[61] Calabrese E., Badea A., Watson C., Johnson G.A. (2013). A quantitative magnetic resonance histology atlas of postnatal rat brain development with regional estimates of growth and variability. Neuroimage.

[62] Semple B.D., Blomgren K., Gimlin K., Ferriero D.M., Noble-Haeusslein L.J. (2013). Brain development in rodents and humans: Identifying benchmarks of maturation and vulnerability to injury across species. Prog. Neurobiol..

[63] Halsted C.H., Medici V., Caballero B., Finglas P.M., Toldrá F. (2016). Alcohol: Metabolism and Health Effects. Encyclopedia of Food and Health.

[64] Papp-Peka A., Tong M., Kril J.J., De La Monte S.M., Sutherland G.T. (2017). The Differential Effects of Alcohol and Nicotine-Specific Nitrosamine Ketone on White Matter Ultrastructure. Alcohol Alcohol..

[65] Wang J.J., Durazzo T.C., Gazdzinski S., Yeh P.H., Mon A., Meyerhoff D.J. (2009). MRSI and DTI: A multimodal approach for improved detection of white matter abnormalities in alcohol and nicotine dependence. NMR Biomed..

[66] Elofson J., Gongvatana W., Carey K.B. (2013). Alcohol use and cerebral white matter compromise in adolescence. Addict. Behav..

[67] Monnig M.A., Tonigan J.S., Yeo R.A., Thoma R.J., McCrady B.S. (2013). White matter volume in alcohol use disorders: A meta-analysis. Addict. Biol..

[68] Lyoo I.K., Streeter C.C., Ahn K.H., Lee H.K., Pollack M.H., Silveri M.M., Nassar L., Levin J.M., Sarid-Segal O., Ciraulo D.A. (2004). White matter hyperintensities in subjects with cocaine and opiate dependence and healthy comparison subjects. Psychiatry Res..

[69] Schulte T., Sullivan E.V., Muller-Oehring E.M., Adalsteinsson E., Pfefferbaum A. (2005). Corpus callosal microstructural integrity influences interhemispheric processing: A diffusion tensor imaging study. Cereb. Cortex.

[70] Ihn Y.K., Hwang S.S., Park Y.H. (2007). Acute Marchiafava-Bignami disease: Diffusion-weighted MRI in cortical and callosal involvement. Yonsei Med. J..

[71] Roux A., Muller L., Jackson S.N., Baldwin K., Womack V., Pagiazitis J.G., O’Rourke J.R., Thanos P.K., Balaban C., Schultz J.A. (2015). Chronic ethanol consumption profoundly alters regional brain ceramide and sphingomyelin content in rodents. ACS Chem. Neurosci..

[72] Yalcin E.B., Nunez K., Tong M., Cornett S.D., de la Monte S.M. (2015). MALDI-IMS Detects Differential White Matter Degeneration-Associated Lipid Profiles in Rat Models of Chronic Alcohol, Tobacco Nitrosamine, or Both Exposures. J. Am. Soc. Mass Spectrom..

[73] Gameiro-Ros I., Noble L., Tong M., Yalcin E., de la Monte S.M. (2023). Tissue Microarray Lipidomic Imaging Mass Spectrometry Method: Application to the Study of Alcohol-Related White Matter Neurodegeneration. Appl. Biosci..

[74] Qu W., Zhang B., Wu D., Xiao B. (1999). Effects of alcohol on membrane lipid fluidity of astrocytes and oligodendrocytes. Wei Sheng Yan Jiu = J. Hyg. Res..

[75] Ingolfsson H.I., Andersen O.S. (2011). Alcohol’s effects on lipid bilayer properties. Biophys. J..

[76] Crews F.T., Nixon K. (2009). Mechanisms of neurodegeneration and regeneration in alcoholism. Alcohol Alcohol..

[77] Schreiber J.A., Tajuddin N.F., Kouzoukas D.E., Kevala K., Kim H.Y., Collins M.A. (2021). Moderate blood alcohol and brain neurovulnerability: Selective depletion of calcium-independent phospholipase A2, omega-3 docosahexaenoic acid, and its synaptamide derivative as a potential harbinger of deficits in anti-inflammatory reserve. Alcohol. Clin. Exp. Res..

[78] Tiwari V., Kuhad A., Chopra K. (2010). Epigallocatechin-3-gallate ameliorates alcohol-induced cognitive dysfunctions and apoptotic neurodegeneration in the developing rat brain. Int. J. Neuropsychopharmacol..

[79] Tiwari V., Chopra K. (2013). Resveratrol abrogates alcohol-induced cognitive deficits by attenuating oxidative-nitrosative stress and inflammatory cascade in the adult rat brain. Neurochem. Int..

[80] Topper L.A., Baculis B.C., Valenzuela C.F. (2015). Exposure of neonatal rats to alcohol has differential effects on neuroinflammation and neuronal survival in the cerebellum and hippocampus. J. Neuroinflamm..

[81] Marcondes M.C., Watry D., Zandonatti M., Flynn C., Taffe M.A., Fox H. (2008). Chronic alcohol consumption generates a vulnerable immune environment during early SIV infection in rhesus macaques. Alcohol. Clin. Exp. Res..

[82] Cippitelli A., Domi E., Ubaldi M., Douglas J.C., Li H.W., Demopulos G., Gaitanaris G., Roberto M., Drew P.D., Kane C.J.M. (2017). Protection against alcohol-induced neuronal and cognitive damage by the PPARgamma receptor agonist pioglitazone. Brain Behav. Immun..

[83] Liu W., Rohlman A.R., Vetreno R., Crews F.T. (2021). Expression of Oligodendrocyte and Oligoprogenitor Cell Proteins in Frontal Cortical White and Gray Matter: Impact of Adolescent Development and Ethanol Exposure. Front. Pharmacol..

[84] Miguel-Hidalgo J.J. (2018). Molecular Neuropathology of Astrocytes and Oligodendrocytes in Alcohol Use Disorders. Front. Mol. Neurosci..

[85] Patro N., Naik A.A., Patro I.K. (2019). Developmental Changes in Oligodendrocyte Genesis, Myelination, and Associated Behavioral Dysfunction in a Rat Model of Intra-generational Protein Malnutrition. Mol. Neurobiol..

[86] Breen M.S., Ozcan S., Ramsey J.M., Wang Z., Ma’ayan A., Rustogi N., Gottschalk M.G., Webster M.J., Weickert C.S., Buxbaum J.D. (2018). Temporal proteomic profiling of postnatal human cortical development. Transl. Psychiatry.

[87] Tong M., Gonzalez-Navarrete H., Kirchberg T., Gotama B., Yalcin E.B., Kay J., de la Monte S.M. (2017). Ethanol-Induced White Matter Atrophy Is Associated with Impaired Expression of Aspartyl-Asparaginyl-beta-Hydroxylase (ASPH) and Notch Signaling in an Experimental Rat Model. J. Drug Alcohol Res..

[88] Campagnoni A.T., Macklin W.B. (1988). Cellular and molecular aspects of myelin protein gene expression. Mol. Neurobiol..

[89] Yang Z., Wang K.K. (2015). Glial fibrillary acidic protein: From intermediate filament assembly and gliosis to neurobiomarker. Trends Neurosci..

[90] Middeldorp J., Hol E.M. (2011). GFAP in health and disease. Prog. Neurobiol..

[91] Osman I., Wang L., Hu G., Zheng Z., Zhou J. (2020). GFAP (Glial Fibrillary Acidic Protein)-Positive Progenitor Cells Contribute to the Development of Vascular Smooth Muscle Cells and Endothelial Cells-Brief Report. Arter. Thromb. Vasc. Biol..

[92] Persson L., Rosengren L. (1977). Increased blood-brain barrier permeability around cerebral stab wounds, aggravated by acute ethanol intoxication. Acta Neurol. Scand..

[93] Rosengren L., Persson L., Johansson B. (1977). Enhanced blood-brain barrier leakage to evans blue-labelled albumin after air embolism in ethanol-intoxicated rats. Acta Neuropathol..

[94] Carrino D., Branca J.J.V., Becatti M., Paternostro F. (2021). Alcohol-Induced Blood-Brain Barrier Impairment: An In Vitro Study. Int. J. Environ. Res. Public Health.

[95] Avchalumov Y., Mandyam C.D. (2020). Synaptic Plasticity and its Modulation by Alcohol. Brain Plast..

[96] Figlia G., Norrmen C., Pereira J.A., Gerber D., Suter U. (2017). Dual function of the PI3K-Akt-mTORC1 axis in myelination of the peripheral nervous system. eLife.

[97] Grier M.D., West K.L., Kelm N.D., Fu C., Does M.D., Parker B., McBrier E., Lagrange A.H., Ess K.C., Carson R.P. (2017). Loss of mTORC2 signaling in oligodendrocyte precursor cells delays myelination. PLoS ONE.

[98] de la Monte S.M., Sutherland G. Dual Stages of Alcohol-Related Cerebral White Matter Degeneration Reviewed: Early-Stage Stress/Neuroinflammation Versus Late-Stage Impaired Insulin/IGF Signaling Through Akt-mTOR–Review.

[99] de la Monte S.M., Wands J.R. (2010). Role of central nervous system insulin resistance in fetal alcohol spectrum disorders. J. Popul. Ther. Clin. Pharmacol..

[100] Biever A., Valjent E., Puighermanal E. (2015). Ribosomal Protein S6 Phosphorylation in the Nervous System: From Regulation to Function. Front. Mol. Neurosci..

[101] Rosner M., Siegel N., Valli A., Fuchs C., Hengstschlager M. (2010). mTOR phosphorylated at S2448 binds to raptor and rictor. Amino Acids.

[102] Liu Y., Ma L., Jiao L., Gao M., Guo W., Chen L., Pan N., Ma Y. (2015). Mammalian target of rapamycin/p70 ribosomal S6 protein kinase signaling is altered by sevoflurane and/or surgery in aged rats. Mol. Med. Rep..

[103] Querfurth H., Lee H.K. (2021). Mammalian/mechanistic target of rapamycin (mTOR) complexes in neurodegeneration. Mol. Neurodegener..

[104] Bockaert J., Marin P. (2015). mTOR in Brain Physiology and Pathologies. Physiol. Rev..

[105] Graber T.E., McCamphill P.K., Sossin W.S. (2013). A recollection of mTOR signaling in learning and memory. Learn. Mem..

[106] Hoeffer C.A., Klann E. (2010). mTOR signaling: At the crossroads of plasticity, memory and disease. Trends Neurosci..

[107] Yang W., Zhou X., Ma T. (2019). Memory Decline and Behavioral Inflexibility in Aged Mice Are Correlated With Dysregulation of Protein Synthesis Capacity. Front. Aging Neurosci..

[108] Zhou X., Lin D.S., Zheng F., Sutton M.A., Wang H. (2010). Intracellular calcium and calmodulin link brain-derived neurotrophic factor to p70S6 kinase phosphorylation and dendritic protein synthesis. J. Neurosci. Res..

[109] Hu Y., Mai W., Chen L., Cao K., Zhang B., Zhang Z., Liu Y., Lou H., Duan S., Gao Z. (2019). mTOR-mediated metabolic reprogramming shapes distinct microglia functions in response to lipopolysaccharide and ATP. Glia.

[110] Laplante M., Sabatini D.M. (2012). mTOR signaling in growth control and disease. Cell.

[111] Harper C.G., Smith N.A., Kril J.J. (1990). The effects of alcohol on the female brain: A neuropathological study. Alcohol Alcohol..

[112] Harada H., Andersen J.S., Mann M., Terada N., Korsmeyer S.J. (2001). p70S6 kinase signals cell survival as well as growth, inactivating the pro-apoptotic molecule BAD. Proc. Natl. Acad. Sci. USA.

[113] Carson R.P., Kelm N.D., West K.L., Does M.D., Fu C., Weaver G., McBrier E., Parker B., Grier M.D., Ess K.C. (2015). Hypomyelination following deletion of Tsc2 in oligodendrocyte precursors. Ann. Clin. Transl. Neurol..

[114] de la Monte S.M., Tong M. (2024). Dysregulated mTOR networks in experimental sporadic Alzheimer’s disease. Front. Cell. Neurosci..

[115] Dudek H., Datta S.R., Franke T.F., Birnbaum M.J., Yao R., Cooper G.M., Segal R.A., Kaplan D.R., Greenberg M.E. (1997). Regulation of neuronal survival by the serine-threonine protein kinase Akt. Science.

[116] de la Monte S.M., Wands J.R. (2005). Review of insulin and insulin-like growth factor expression, signaling, and malfunction in the central nervous system: Relevance to Alzheimer’s disease. J. Alzheimer’s Dis. JAD.

[117] Yoon M.S. (2017). The Role of Mammalian Target of Rapamycin (mTOR) in Insulin Signaling. Nutrients.

[118] Myers M.G., Sun X.J., White M.F. (1994). The IRS-1 signaling system. Trends Biochem. Sci..

[119] White M.F. (2002). IRS proteins and the common path to diabetes. Am. J. Physiol. Endocrinol. Metab..

[120] Schmitz-Peiffer C., Whitehead J.P. (2003). IRS-1 regulation in health and disease. IUBMB Life.

[121] Schubert M., Brazil D.P., Burks D.J., Kushner J.A., Ye J., Flint C.L., Farhang-Fallah J., Dikkes P., Warot X.M., Rio C. (2003). Insulin receptor substrate-2 deficiency impairs brain growth and promotes tau phosphorylation. J. Neurosci..

[122] Freude S., Leeser U., Muller M., Hettich M.M., Udelhoven M., Schilbach K., Tobe K., Kadowaki T., Kohler C., Schroder H. (2008). IRS-2 branch of IGF-1 receptor signaling is essential for appropriate timing of myelination. J. Neurochem..

[123] Copps K.D., White M.F. (2012). Regulation of insulin sensitivity by serine/threonine phosphorylation of insulin receptor substrate proteins IRS1 and IRS2. Diabetologia.

[124] Morino K., Petersen K.F., Dufour S., Befroy D., Frattini J., Shatzkes N., Neschen S., White M.F., Bilz S., Sono S. (2005). Reduced mitochondrial density and increased IRS-1 serine phosphorylation in muscle of insulin-resistant offspring of type 2 diabetic parents. J. Clin. Investig..

[125] Pearl L.H., Barford D. (2002). Regulation of protein kinases in insulin, growth factor and Wnt signalling. Curr. Opin. Struct. Biol..

[126] Hers I., Vincent E.E., Tavare J.M. (2011). Akt signalling in health and disease. Cell. Signal..

[127] Lindtner C., Scherer T., Zielinski E., Filatova N., Fasshauer M., Tonks N.K., Puchowicz M., Buettner C. (2013). Binge drinking induces whole-body insulin resistance by impairing hypothalamic insulin action. Sci. Transl. Med..

[128] Xu J., Yeon J.E., Chang H., Tison G., Chen G.J., Wands J., de la Monte S. (2003). Ethanol impairs insulin-stimulated neuronal survival in the developing brain: Role of PTEN phosphatase. J. Biol. Chem..

[129] de la Monte S.M., Wands J.R. (2002). Chronic gestational exposure to ethanol impairs insulin-stimulated survival and mitochondrial function in cerebellar neurons. Cell. Mol. Life Sci..

[130] Bercury K.K., Dai J., Sachs H.H., Ahrendsen J.T., Wood T.L., Macklin W.B. (2014). Conditional ablation of raptor or rictor has differential impact on oligodendrocyte differentiation and CNS myelination. J. Neurosci..

[131] Rosner M., Hengstschlager M. (2008). Cytoplasmic and nuclear distribution of the protein complexes mTORC1 and mTORC2: Rapamycin triggers dephosphorylation and delocalization of the mTORC2 components rictor and sin1. Hum. Mol. Genet..

[132] Cai S.L., Tee A.R., Short J.D., Bergeron J.M., Kim J., Shen J., Guo R., Johnson C.L., Kiguchi K., Walker C.L. (2006). Activity of TSC2 is inhibited by AKT-mediated phosphorylation and membrane partitioning. J. Cell Biol..

[133] Matsuda T., Zhai P., Maejima Y., Hong C., Gao S., Tian B., Goto K., Takagi H., Tamamori-Adachi M., Kitajima S. (2008). Distinct roles of GSK-3alpha and GSK-3beta phosphorylation in the heart under pressure overload. Proc. Natl. Acad. Sci. USA.

[134] Zhou J., Freeman T.A., Ahmad F., Shang X., Mangano E., Gao E., Farber J., Wang Y., Ma X.L., Woodgett J. (2013). GSK-3alpha is a central regulator of age-related pathologies in mice. J. Clin. Investig..

[135] Kaidanovich-Beilin O., Lipina T.V., Takao K., van Eede M., Hattori S., Laliberte C., Khan M., Okamoto K., Chambers J.W., Fletcher P.J. (2009). Abnormalities in brain structure and behavior in GSK-3alpha mutant mice. Mol. Brain.

[136] Pavlov D., Markova N., Bettendorff L., Chekhonin V., Pomytkin I., Lioudyno V., Svistunov A., Ponomarev E., Lesch K.P., Strekalova T. (2017). Elucidating the functions of brain GSK3alpha: Possible synergy with GSK3beta upregulation and reversal by antidepressant treatment in a mouse model of depressive-like behaviour. Behav. Brain Res..

[137] Gulen M.F., Bulek K., Xiao H., Yu M., Gao J., Sun L., Beurel E., Kaidanovich-Beilin O., Fox P.L., DiCorleto P.E. (2012). Inactivation of the enzyme GSK3alpha by the kinase IKKi promotes AKT-mTOR signaling pathway that mediates interleukin-1-induced Th17 cell maintenance. Immunity.

[138] Doble B.W., Woodgett J.R. (2003). GSK-3: Tricks of the trade for a multi-tasking kinase. J Cell Sci.

[139] Carter J.J., Tong M., Silbermann E., Lahousse S.A., Ding F.F., Longato L., Roper N., Wands J.R., de la Monte S.M. (2008). Ethanol impaired neuronal migration is associated with reduced aspartyl-asparaginyl-beta-hydroxylase expression. Acta Neuropathol..

[140] Vincent T., Kukalev A., Andang M., Pettersson R., Percipalle P. (2008). The glycogen synthase kinase (GSK) 3beta represses RNA polymerase I transcription. Oncogene.

[141] Luo J. (2010). Lithium-mediated protection against ethanol neurotoxicity. Front. Neurosci..

[142] He J., de la Monte S., Wands J.R. (2007). Acute ethanol exposure inhibits insulin signaling in the liver. Hepatology.

[143] Hermida M.A., Dinesh Kumar J., Leslie N.R. (2017). GSK3 and its interactions with the PI3K/AKT/mTOR signalling network. Adv Biol. Regul..

[144] Noori T., Dehpour A.R., Sureda A., Fakhri S., Sobarzo-Sanchez E., Farzaei M.H., Kupeli Akkol E., Khodarahmi Z., Hosseini S.Z., Alavi S.D. (2020). The role of glycogen synthase kinase 3 beta in multiple sclerosis. Biomed. Pharmacother..

[145] Toral-Rios D., Pichardo-Rojas P.S., Alonso-Vanegas M., Campos-Pena V. (2020). GSK3beta and Tau Protein in Alzheimer’s Disease and Epilepsy. Front. Cell. Neurosci..

[146] Akhtar A., Sah S.P. (2020). Insulin signaling pathway and related molecules: Role in neurodegeneration and Alzheimer’s disease. Neurochem. Int..

[147] Luo J. (2009). GSK3beta in ethanol neurotoxicity. Mol. Neurobiol..

[148] Takashima A. (2006). GSK-3 is essential in the pathogenesis of Alzheimer’s disease. J. Alzheimer’s Dis. JAD.

[149] Xiong T., Qu Y., Wang H., Chen H., Zhu J., Zhao F., Zou R., Zhang L., Mu D. (2018). GSK-3beta/mTORC1 Couples Synaptogenesis and Axonal Repair to Reduce Hypoxia Ischemia-Mediated Brain Injury in Neonatal Rats. J. Neuropathol. Exp. Neurol..

[150] Liu R.J., Fuchikami M., Dwyer J.M., Lepack A.E., Duman R.S., Aghajanian G.K. (2013). GSK-3 inhibition potentiates the synaptogenic and antidepressant-like effects of subthreshold doses of ketamine. Neuropsychopharmacology.

[151] Krishnankutty A., Kimura T., Saito T., Aoyagi K., Asada A., Takahashi S.I., Ando K., Ohara-Imaizumi M., Ishiguro K., Hisanaga S.I. (2017). In vivo regulation of glycogen synthase kinase 3beta activity in neurons and brains. Sci. Rep..

[152] Skelton P.D., Stan R.V., Luikart B.W. (2020). The Role of PTEN in Neurodevelopment. Mol. Neuropsychiatry.

[153] van Diepen M.T., Eickholt B.J. (2008). Function of PTEN during the formation and maintenance of neuronal circuits in the brain. Dev. Neurosci..

[154] Mukherjee R., Vanaja K.G., Boyer J.A., Gadal S., Solomon H., Chandarlapaty S., Levchenko A., Rosen N. (2021). Regulation of PTEN translation by PI3K signaling maintains pathway homeostasis. Mol. Cell.

[155] Bhattacharya K., Maiti S., Mandal C. (2016). PTEN negatively regulates mTORC2 formation and signaling in grade IV glioma via Rictor hyperphosphorylation at Thr1135 and direct the mode of action of an mTORC1/2 inhibitor. Oncogenesis.

[156] Vazquez F., Grossman S.R., Takahashi Y., Rokas M.V., Nakamura N., Sellers W.R. (2001). Phosphorylation of the PTEN tail acts as an inhibitory switch by preventing its recruitment into a protein complex. J. Biol. Chem..

[157] Vazquez F., Ramaswamy S., Nakamura N., Sellers W.R. (2000). Phosphorylation of the PTEN tail regulates protein stability and function. Mol. Cell. Biol..

[158] Bartolome A., Kimura-Koyanagi M., Asahara S., Guillen C., Inoue H., Teruyama K., Shimizu S., Kanno A., Garcia-Aguilar A., Koike M. (2014). Pancreatic beta-cell failure mediated by mTORC1 hyperactivity and autophagic impairment. Diabetes.

[159] Huang J., Dibble C.C., Matsuzaki M., Manning B.D. (2008). The TSC1-TSC2 complex is required for proper activation of mTOR complex 2. Mol. Cell. Biol..

[160] Orlova K.A., Crino P.B. (2010). The tuberous sclerosis complex. Ann. N. Y. Acad. Sci..

[161] Inoki K., Li Y., Xu T., Guan K.L. (2003). Rheb GTPase is a direct target of TSC2 GAP activity and regulates mTOR signaling. Genes Dev..

[162] Inoki K., Li Y., Zhu T., Wu J., Guan K.L. (2002). TSC2 is phosphorylated and inhibited by Akt and suppresses mTOR signalling. Nat. Cell Biol..

[163] Agarwal S., Bell C.M., Rothbart S.B., Moran R.G. (2015). AMP-activated Protein Kinase (AMPK) Control of mTORC1 Is p53- and TSC2-independent in Pemetrexed-treated Carcinoma Cells. J. Biol. Chem..

[164] Inoki K., Ouyang H., Zhu T., Lindvall C., Wang Y., Zhang X., Yang Q., Bennett C., Harada Y., Stankunas K. (2006). TSC2 integrates Wnt and energy signals via a coordinated phosphorylation by AMPK and GSK3 to regulate cell growth. Cell.

[165] Tian T., Li X., Zhang J. (2019). mTOR Signaling in Cancer and mTOR Inhibitors in Solid Tumor Targeting Therapy. Int. J. Mol. Sci..

[166] Lee D.Y. (2015). Roles of mTOR Signaling in Brain Development. Exp. Neurobiol..

[167] Ishizuka Y., Kakiya N., Nawa H., Takei N. (2008). Leucine induces phosphorylation and activation of p70S6K in cortical neurons via the system L amino acid transporter. J. Neurochem..

[168] Copp J., Manning G., Hunter T. (2009). TORC-specific phosphorylation of mammalian target of rapamycin (mTOR): Phospho-Ser2481 is a marker for intact mTOR signaling complex 2. Cancer Res..

[169] Chiang G.G., Abraham R.T. (2005). Phosphorylation of mammalian target of rapamycin (mTOR) at Ser-2448 is mediated by p70S6 kinase. J. Biol. Chem..

[170] Kyriakis J.M., Avruch J. (2010). Insulin and Growth Factor Signaling Pathways. Endocrinology, Jameson, J.L., De Groot, L.J., Eds..

[171] Rosner M., Schipany K., Hengstschlager M. (2012). p70 S6K1 nuclear localization depends on its mTOR-mediated phosphorylation at T389, but not on its kinase activity towards S6. Amino Acids.

[172] Oddo S. (2015). The Mtor/P70s6k Pathway Plays a Key Role in the Pathogenesis of Alzheimer’s Disease. Gerontologist.

[173] Acosta-Jaquez H.A., Keller J.A., Foster K.G., Ekim B., Soliman G.A., Feener E.P., Ballif B.A., Fingar D.C. (2009). Site-specific mTOR phosphorylation promotes mTORC1-mediated signaling and cell growth. Mol. Cell. Biol..

[174] Arif A., Jia J., Willard B., Li X., Fox P.L. (2019). Multisite Phosphorylation of S6K1 Directs a Kinase Phospho-code that Determines Substrate Selection. Mol. Cell.

[175] de la Monte S.M., Tong M., Schiano I., Didsbury J. (2017). Improved Brain Insulin/IGF Signaling and Reduced Neuroinflammation with T3D-959 in an Experimental Model of Sporadic Alzheimer’s Disease. J. Alzheimer’s Dis. JAD.

[176] Nguyen V.A., Le T., Tong M., Silbermann E., Gundogan F., de la Monte S.M. (2012). Impaired insulin/IGF signaling in experimental alcohol-related myopathy. Nutrients.

[177] Lu T., Zhu Z., Wu J., She H., Han R., Xu H., Qin Z.H. (2019). DRAM1 regulates autophagy and cell proliferation via inhibition of the phosphoinositide 3-kinase-Akt-mTOR-ribosomal protein S6 pathway. Cell Commun. Signal..

[178] Kim D.H., Sarbassov D.D., Ali S.M., King J.E., Latek R.R., Erdjument-Bromage H., Tempst P., Sabatini D.M. (2002). mTOR interacts with raptor to form a nutrient-sensitive complex that signals to the cell growth machinery. Cell.

[179] Liu L., Luo Y., Chen L., Shen T., Xu B., Chen W., Zhou H., Han X., Huang S. (2010). Rapamycin inhibits cytoskeleton reorganization and cell motility by suppressing RhoA expression and activity. J. Biol. Chem..

[180] Gwinn D.M., Shackelford D.B., Egan D.F., Mihaylova M.M., Mery A., Vasquez D.S., Turk B.E., Shaw R.J. (2008). AMPK phosphorylation of raptor mediates a metabolic checkpoint. Mol. Cell.

[181] Garza-Lombo C., Schroder A., Reyes-Reyes E.M., Franco R. (2018). mTOR/AMPK signaling in the brain: Cell metabolism, proteostasis and survival. Curr. Opin. Toxicol..

[182] Sarbassov D.D., Ali S.M., Kim D.H., Guertin D.A., Latek R.R., Erdjument-Bromage H., Tempst P., Sabatini D.M. (2004). Rictor, a novel binding partner of mTOR, defines a rapamycin-insensitive and raptor-independent pathway that regulates the cytoskeleton. Curr. Biol..

[183] Kocalis H.E., Hagan S.L., George L., Turney M.K., Siuta M.A., Laryea G.N., Morris L.C., Muglia L.J., Printz R.L., Stanwood G.D. (2014). Rictor/mTORC2 facilitates central regulation of energy and glucose homeostasis. Mol. Metab..

[184] Thomanetz V., Angliker N., Cloetta D., Lustenberger R.M., Schweighauser M., Oliveri F., Suzuki N., Ruegg M.A. (2013). Ablation of the mTORC2 component rictor in brain or Purkinje cells affects size and neuron morphology. J. Cell Biol..

[185] Julien L.A., Carriere A., Moreau J., Roux P.P. (2010). mTORC1-activated S6K1 phosphorylates Rictor on threonine 1135 and regulates mTORC2 signaling. Mol. Cell. Biol..

