# Supplementary material for: Progressive Alcohol-Related Brain Atrophy and White Matter Pathology Are Linked to Long-Term Inhibitory Effects on mTOR Signaling
Source: Biomolecules. 2025 Mar 14;15(3):413. doi: 10.3390/biom15030413 (PMC11940526; doi:10.3390/biom15030413)
Supplement: Supplementary file 1 [file biomolecules-15-00413-s001.zip › biomolecules-3485786-supplementary.pdf]

Supplementary Table S1: Critical Reagent and Instrument Sources

**Reagents**	**Commercial Source**
BCA assay, secondary HRP-conjugated antibodies, Superblock-TBS, MaxiSorp 96-well plates	Thermo Fisher Scientific, Bedford MA USA
Amplex UltraRed soluble fluorophore, 4-Methylumbelliferyl phosphate	Life Technologies, Carlsbad, CA USA
Alkaline phosphatase streptavidin, Proton Biotin Protein Labeling Kit	Vector Laboratories Inc, Newark CA USA
Total and phospho-Akt/mTOR Multiplex panels	Millipore-Sigma, Bedford, MA USA
5-Plex MILLIPLEX MAP Rat Cytokine Magnetic Bead Panel	Millipore-Sigma, Bedford, MA USA
Lieber-deCarli Liquid Diets	Bio-Serv, Flemington, NJ USA
Ethanol Colorimetric/Fluorometric Assay Kit (BioVision)	Avantor, Bridgeport, NJ USA
Instruments	Commercial Source
Luminex MAGPIX	Luminex Corp, Austin TX USA
SpectraMax M5 microplate reader	Molecular Devices, San Jose, CA USA
TissueLyser II	Qiagen, Germantown, MD USA

Supplementary Table S2: Antibody Sources and Preparations

Antibody Name	Antibody Type	Stock	Final Concentration	Commercial Source	Catalogue #	RRID
CNPase (11-5B)	Monoclonal-Mouse	1 mg/mL	2 µg/mL	Abcam	ab6319	AB_2082593
PLP	Polyclonal-Rabbit	Purity: whole antiserum	1:2000	Abcam	ab28486	AB_776593
PDGFRA	Monoclonal-Rabbit	0.615 mg/mL	1.23 µg/mL	Abcam	AB203491	AB_2892065
Galc	Monoclonal-Rabbit	0.585 mg/mL	1.17 µg/mL	Abcam	ab240638	AB_3101785
MAG1	Monoclonal-Mouse	0.5 mg/mL	0.5 µg/mL	Abcam	ab89780	AB_2042411
MOG	Polyclonal-Rabbit	1 mg/mL	2 µg/mL	Sigma	M3821	AB_2145529
MBP	Polyclonal-Rabbit	1 mg/mL	2 µg/mL	Abcam	ab32760	AB_1841021
Nestin	Monoclonal-Mouse	1 mg/mL	1 µg/mL	Abcam	ab6142	AB_305313
Vimentin (RV202)	Monoclonal-Mouse	1 mg/mL	2.5 µg/mL	Abcam	ab8978	AB_306907
GFAP	Polyclonal-Goat	0.5 mg/mL	0.25 µg/mL	Abcam	ab53554	AB_880202
Rictor	Polyclonal-Rabbit	1 mg/mL	0.33 µg/mL	Invitrogen (Thermo Fisher Scientific	PA5-102842	AB_2852231
Phospho Rictor(Ser1591)	Polyclonal-Rabbit	1 mg/mL	2.0 µg/mL	Invitrogen (Thermo Fisher Scientific	PA5-105669	AB_2817097

Raptor(14C4)	Monoclonal-Mouse	1 mg/mL	1.25 µg/mL	Bioss (Thermo Fisher Scientific	BSM-51285M	AB_2897588
Phospho Raptor(Ser792)	Polyclonal-Rabbit	1 mg/mL	0.5 µg/mL	Bioss (Thermo Fisher Scientific	BS-3381R	AB_10883949
RPLPO	Monoclonal-Mouse	0.1 mg/mL	0.1 µg/mL	Santa Cruz Biotechnology	sc-293260	RPL23 16086-1-AP

Supplementary Table S3A: 11-Plex Akt-mTOR Pathway (Proteins and Phosphoproteins)

Akt/mTOR Pathway Molecules	Protein Abbreviation	Phospho-protein
Insulin Receptor	Insulin-R	$^{pYpY1162/1163}$-Insulin R
Insulin-Like Growth Factor Receptor Type 1	IGF-1R	$^{pYpY1135/1136}$-IGF-1R
Insulin Receptor Substrate, Type 1	IRS1	^{pS636}-IRS-1
Akt (Protein Kinase B)	Akt	^{pS473}-Akt
Phosphatase and tensin homolog	PTEN	^{pS380}-PTEN
Glycogen Synthase Kinase 3α	GSK-3α	^{pS21}-GSK3α
Glycogen Synthase Kinase 3β	GSK-3β	^{pS9}-GSK3β
p70 Ribosomal S6 kinase	P70S6K	^{pT412}-p70S6K
Ribosomal Protein S6	RPS6	$^{pS235/S236}$-RPS6
Mechanistic Target of Rapamycin	mTOR	^{pS2448}-mTOR
Tuberous Sclerosis Complex 2	TSC2	^{pS939}-TSC2

Supplementary Table S3B: Rat Cytokine 5-Plex Antibodies

Cytokines	Abbreviation
Interleukin-1β	IL-1β
Interleukin-2	IL-2
Interleukin-6	IL-6
Interferon-γ	IFN-γ
Tumor Necrosis Factor-α	TNF-α

Supplementary Table S4: mTOR Signaling Proteins and Brain Functions

Abbreviation	Full Name	Functions	Brain Functions in relation to mTOR
Insulin R	Insulin Receptor	Insulin receptor signaling mediates cell survival, metabolism, and proliferation [115,116].	Insulin receptor signaling through IRS1 activates mTORC1 [117]
$^{pYpY1162/1163}$-Insulin R	Tyrosine phosphorylated insulin receptor; receptor tyrosine kinase activated	Tyrosine phosphorylation of the insulin receptor activates the intrinsic receptor tyrosine kinase leading to increased phosphatidylinositol-3-kinase (PI3K) activity through phosphorylation of adaptors, such as the insulin receptor substrate 1 (IRS1) [115,116]	Insulin receptor tyrosine kinase-mediated tyrosine phosphorylation IRS1 activates mTOR. mTOR controls insulin signaling by negative feedback via serine phosphorylation of the insulin receptor [117].
IGF-1R	Insulin-like growth factor type 1 receptor	IGF-1 receptor signaling mediates neuronal and oligodendrocyte growth and proliferation and oligodendrocyte myelination [115,116].	IGF-1 signaling activates mTORC1 via receptor tyrosine kinase-mediated tyrosine phosphorylation of IRS1 [117].
$^{pYpY1135/1136}$-IGF-1R	Tyrosine phosphorylated IGF-1 receptor—receptor tyrosine kinase activated	Tyrosine phosphorylation of the IGF-1 receptor activates the intrinsic receptor tyrosine kinase leading to increased phosphatidylinositol-3-kinase (PI3K) activity through phosphorylation of adaptors, such as the insulin receptor substrate 1 (IRS1) [115,116]	IGF-1R tyrosine kinase mediates tyrosine phosphorylation IRS1 to activate mTOR. mTOR controls IGF-1R signaling by negative feedback via serine phosphorylation of the insulin receptor [117]
IRS-1	Insulin receptor substrate, type 1	Insulin receptor substrate proteins are major multifaceted docking molecules for transmitting complex signals downstream from cell surface receptors to intracellular cascades that mediate a broad array of functions [118-120]. Although both IRS1 and IRS2 are expressed in the brain, IRS2's effects are dominant with respect to neuronal and oligodendrocyte functions [121,122].	Insulin or IGF-1 receptor mediated tyrosine phosphorylation of IRS-1 followed by increased PI3K-then PDK1 which phosphorylates TSC1/2 and PRAS40, dis-inhibiting formation of the mTORC1 complex and activation of mTORC1→S6K1 [117].
^{pS636}-IRS-1	Serine-636 phosphorylated IRS-1- functions inhibited	IRS1 and IRS2 are regulated by Tyrosine (positive) and Serine (negative) phosphorylation [123].Ser -636 phosphorylation of IRS1 is associated with insulin resistance and mitochondrial dysfunction [124].	mTORC1 provides negative feedback on IRS1 signaling by promoting its serine phosphorylation and subsequent degradation.

Akt	Protein Kinase B	Akt is a downstream target of growth factor stimulation including via insulin and IGF-1 receptor signaling, and has important downstream regulatory roles in cell growth, metabolism, and survival [125,126]. In addition, Akt has an important role in white matter oligodendrocyte myelination [123]	Akt stimulates myelination via mTOR signaling [30,31] and mediates its effects on cell growth by phosphorylating TSC2. The resulting destabilization of TSC2 protein releases the check on Ras homolog enriched in brain (RHEB), enabling activation of mTOR/mTORC1 [117].
^{pS473}-Akt	Serine-473 phosphorylated Akt-with kinase activated	Serine phosphorylation of Akt activates its kinase, which is needed to mediate growth, survival and metabolic signaling. Inhibition of Akt phosphorylation and kinase activity correlate with reduced neuronal and oligodendrocyte functions, as occurs in the brain with ethanol neurotoxicity [18,99,127-129].	Indirectly activates mTORC by phosphorylating and inhibiting TSC1/2 as well as PRAS40 [117] The levels of pS473-Akt correspond to mTORC2 activation of the kinase [130]. PI3K-PDK1 activates Akt via T308 phosphorylation. Activated Akt phosphorylates TSC1/2 at S939 and T1462 leading to downregulation of the GTPase activating (GAP) potential of tuberin, the gene product of the TSC2 and inhibition of RHEB, a potent regulator of the mTOR signaling [131,132].
GSK-3α	Glycogen synthase kinase-3α	GSK-3α localizes in the nucleus, down-regulates G1 cyclins, suppresses E2F and markers of cell proliferation [133]. Protects against aging-associated pathologies [134].	Roles in neurobehavioral/psychiatric function [135] including depression [136]. Role in motor function, coordination, exploratory behavior, sensorimotor memory coordination [135].
^{pS21}-GSK3α	Serine-21 phosphorylated GSK-3α —with kinase activity inhibited	Kinase activity is inactivated by Ser-21 phosphorylation of GSK-3α [133]. Research that distinguishes specific functions of GSK-3α from GSK-3β are limited.	A critical signaling intermediate phosphorylated and inhibited by IL-1 activation of IKKi, leading to Akt activation of mTOR [137].
GSK-3β	Glycogen synthase kinase-3β	Ser/Thr protein kinase, ubiquitously expressed in the CNS and master regulator of signaling through multiple pathways including Canonical Wnt (β-catenin), Notch, RTK, G-protein coupled receptor and sonic hedgehog [125,138]. Negative effect on axon formation, neuronal migration, and cytoskeleton regulation [139]. Targets PTEN for negative feedback on Akt via phosphorylation [18,129,140,141]. Regulates neurogenesis, synaptic transmission, and	Phosphorylates upstream and downstream molecules in the Akt-mTOR network including Akt, Rictor, TSC1/2, PTEN, and IRS [143]. Inhibits signaling functions of proteins that regulate Canonical Wnt (β-catenin), Notch, RTK, G-protein coupled receptor and sonic hedgehog [125,138]. GSK-3β is up-regulated by alcohol's neurotoxic effects and neurodegeneration [144-148]. GSK-3β inhibits mTORC1 expression of

		plasticity. Increased by alcohol exposure [19,142].	synaptic proteins and neuronal proteins needed for regeneration after injury [149,150].
^{pS9}-GSK-3β	Serine-9 phosphorylated GSK-3β with kinase activity inhibited	Kinase activity is inactivated by Ser-9 phosphorylation of GSK-3β [125]. Ser-9 phosphorylation by Akt, PKA, and P90RSK inhibit its constitutively active kinase activity [151].	Inhibition of GSK-3β via phosphorylation has a critical role in regulating cellular responses to growth factors including insulin and IGF-1 receptor signal transduction through IRS1-PI3K-Akt-mTOR [143].
PTEN	Phosphatase and tensin homolog	Lipid and protein phosphatase that negatively regulates cell growth, proliferation, motility, survival, and plasticity, and promotes apoptosis via inhibition of PI3K and Akt [19,126,152,153]. Loss or mutation of PTEN enhances neuronal proliferation, migration, survival, plasticity, morphology [152]. PTEN inhibition of MAPK compromises neurite outgrowth and cause growth cone collapse when localized in growth cones. Alcohol increases PTEN expression and function [128,142].	Upstream negative regulator of mTOR due to its inhibitory effects on PI3K-Akt activation of mTORC1 . PI3K signaling increases PTEN expression as part of a negative feedback mechanism. PTEN is transcriptionally regulated by mTOR/4E-BP1[154]. PTEN regulates mTORC2 by targeting Rictor for Thr1135 hyperphosphorylation, leading to its reduced association with mTOR [155].
^{pS380}-PTEN	Serine-380 phosphorylated PTEN—extend the half-life by reducing its degradation	Phosphorylation by CK2 kinase slows PTEN degradation by the proteasome but also reduces membrane interactions, reflecting an inhibitor switch for PTEN [156,157]. Lipid-binding of the C2 domain promotes phosphorylation blocks or suppresses the phosphatase activity [156,157].	GSK-3β phosphorylates PTEN [143]. Phosphorylation of the PTEN tail leads to stabilization of the protein but also inhibits PTEN's function [157] by preventing its interactions with PDZ domain-containing proteins [156]. Inhibition of PTEN by phosphorylation would likely increase mTOR activation.
TSC2	Tuberous sclerosis complex protein 2	Tumor suppressor gene that encodes tuberin, a growth inhibitory protein (upstream regulator of mTOR). Tuberin's interaction with hamartin forms TSC protein complex that functions in cell growth control. TSC2 functions within a multi-protein complex (TSC), including TSC2, TSC1, and TBC1D1. TSC1 and TSC2 establish heterodimer complex. TSC1 has a major role in stabilizing TSC2 and preventing its ubiquitin-mediated proteasomal degradation [158]. TSC1-TSC2	TSC1 and TSC2 modulate via mTOR signaling with outcomes related to cell body size, dendritic arborization, axonal outgrowth, neuronal migration, cortical lamination [160]. Insulin and IGF-1 signaling inhibit TSC2:TSC1 complex via Akt phosphorylation which inactivates TSC2. Non-phosphorylated TSC2 inhibits insulin stimulated S6K. Tuberin's interaction with hamartin forms TSC protein complex that negatively regulates mTORC1 signaling, a major regulator of anabolic cell growth. TSC complex brakes mTORC1 activity via Ras homolog

		complex physically associates with mTORC2 and activates it, promoting Akt activation, but the complex inhibits TSC1 [159].	enriched in brain (RHEB) GTPase activity causing RHEB-GTP hydrolysis to RHEB-GDP to shut off mTORC1. AMPK and GSK3 activate TSC2:TSC1 via phosphorylation at Thr1227 and Ser1345 which is needed for negative feedback inhibition of S6K in response to energy stress/depletion [160-162].
^{pS939}-TSC2	Serine-939 phosphorylated TSC2-inactivated/ inhibited	Ser-939 phosphorylation negatively regulates and inhibits the function of TSC2. TSC2 phosphorylation is regulated by insulin signaling via Akt as well as ERK1/2 [158,161,162]. AMPK phosphorylation of TSC2 does the opposite and stabilizes the TSC1-TSC2 complex [161,163]. Like AMPK, Wnt phosphorylation of TSC2 via GSK-3β enhances TSC2 stability and function. [164].	**Akt and ERK1/2** phosphorylation of TSC2 enables mTORC1 activation [158,161,162]. ROS or hypoxia downregulate mTORC1 signaling by increasing GSK-3b activation, which in the brain leads to increased synaptogenesis [149]. AMPK phosphorylation of TSC2 on Ser-1387 does the opposite and stabilizes the TSC1-TSC2 complex resulting in decreased mTORC1 [163]. Wnt phosphorylates TSC2 via GSK-3β, working with AMPK to brake mTORC1 [164].
mTOR	Mammalian or Mechanistic target of rapamycin	mTOR signaling is mediated via two main complexes termed mTORC1 and mTORC2; mTOR is at the center of both. The mTORC1 complex, which is inhibited by rapamycin, is composed of mTOR complexed with Raptor, TSC, mLST8, PRAS40, and DEPTOR and localized in endosomal and lysosomal membranes [101,165]. mTORC2, which is insensitive to rapamycin, is composed of mTOR bound to Rictor, Protor-1/2, mammalian stress-activated MAP kinase-interacting protein 1 (mSIN1), mLST8 and DEPTOR [104] and associated with the plasma and ribosomal membranes [165]. mTOR signaling is responsive to cellular nutrition, energy levels and growth factor stimulation. mTORC1/2 has broad cellular effects, promoting mitochondrial function, cytoskeletal organization, cell motility/migration, dendrite formation, glial differentiation, and lipid and protein metabolism while inhibiting autophagy [101]. In glia, mTOR has roles in	mTOR-controlled signaling pathways regulate many integrated physiological functions of the nervous system including neuronal development, synaptic plasticity, memory storage, and cognition. Deregulation of mTOR signaling is associated with many neurological and psychiatric disorders [104], most likely due to the effects on axonal sprouting, axonal regeneration, myelination, dendritic spine growth [104,130] . mTORC1 signaling is enabled by the phosphorylation and attendant inhibition of TSC1-TSC2 [162] or PRAS40, releasing their control on mTOR/ mTORC1 signaling [104]. The end result is stimulation of cellular proliferation, growth, RNA translation, and nutrient metabolism [166], and inhibition of autophagy [158]. Akt phosphorylates and activates mTORC2 [131,101] and has a role in synaptic plasticity [167].

		myelination and clearing extracellular glutamate, and in neurons it regulates synaptic functions. mTORC1 regulates astrocyte differentiation. TORC2 has been implicated in the regulation of actin cytoskeleton organization and has been shown to activate Akt. [160] and S6K. Hyperactivation of Rictor-containing mTORC2 increases brain gliogenesis [166].	
^{pS2448}-mTOR	Serine-2448 phosphorylated mTOR-Activated	Serine phosphorylation of mTOR marks its activated state. mTOR phosphorylated at Ser-2448 complexes in mTORC1 whereas mTOR phosphorylated at Ser2481 interacts with mTORC2 [168. Ser2448 and Ser2481 respectively mark mTORC1 and mTORC2 kinase activation [168,169].	^{pS2448}-mTOR binds to Raptor and Rictor while ^{pS2481}-mTOR binds to Rictor to activate mTORC1/mTORC2 signaling [101,168]. ^{pS2448}-mTOR increases lipogenesis, lipid storage [117]. Activation of mTOR signaling regulates neuronal and glial development, synaptic plasticity, memory, cognition, behavior [104,166], myelination [30,31,113].
P70S6K	P70S6K protein	P70S6K is a cytoplasmic Ser/Thr kinase that regulates RSP6 through phosphorylation. P70S6K regulates mRNA translation, modulates cell cycle progression, cell survival, and cell size. Mitogen stimulation of P70S6K such as with IGF-1, enhances translation by increasing ribosome biosynthesis, and promotes anti-apoptotic and pro-survival mechanisms. Its supportive effects on cell survival/anti-apoptosis are mediated by inhibition of mitochondrial BAD via phosphorylation and activation of RS6 [112].	P70S6K is a major substrate for Akt activated mTORC1 [101]. TSC1-TSC2 inhibits mTORC1 activation of P70S6K [162].
^{pT412}-p70S6K	Activated p70S6K	mTORC1 kinase phosphorylates p70S6K on T412 within its catalytic domain extension and activates its kinase [170]. mTORC phosphorylation of p70S6K at T389 drives its nuclear localization [171]. Leucine activates p70S6K in cortical neurons [167] and mediates tau synthesis and phosphorylation. Consequently, Leu-mediated phosphorylation and hyper-activation of p70S6K is considered	S1261 phosphorylation of mTORC1 promotes mTORC1-mediated phosphorylation of p70S6K as well as of the eukaryotic initiation factor 4E binding protein 1 (4E-BP1) to upregulate protein synthesis [173]. Phosphorylated/activated p70S6K targets RS6K, phosphorylating and activating the protein kinase at S235 and S236 [174], and thus marking activation of mTORC1 signaling [168,169]. Inhibition of p70S6K activity is associated with experimental Alzheimer-type

		potentially pathogenic in Alzheimer's disease [172].	and alcohol-mediated neurodegeneration and neurodevelopmental pathologies [19,175,176].
RSP6	Ribosomal SP6 Kinase protein	The 40S ribosomal protein kinase, RPS6K, upregulates protein synthesis, enhances lipid synthesis, mitochondrial biogenesis, and inhibits 4E-BP1 which inhibits protein synthesis and inhibits autophagy [100,177].	mTOR/mTORC1 mediates RSP6's activated functions in regulating cell size, growth, proliferation [100], and synaptic plasticity.<br>Independent of mTOR, TSC1-TSC2 suppresses S6 kinase's inhibition by PI3 Kinase.
$^{pS235/236}$-RPS6	Activated RPS6 Kinase	$^{pS235/236}$-RPS6 is the active form of S6 and functional readout of mTORC1 activation of p70S6K. $^{pS235/236}$-RPS6 provides negative feedback to IRS1-PI3K-Akt. Protein phosphatase 1 (PP-1) dephosphorylates and inactivates $^{pS235/236}$-RPS6 [100].	Active form of S6; negative; used as readout of mTORC1 signaling activation or marker of neuronal activity [100] and increased with synaptic plasticity [100].
Raptor	Regulatory-associated protein of mTOR	Raptor is a critical adaptor protein in the mTOR complex 1 (mTORC1) signaling pathway, regulating cell growth, metabolism, and protein synthesis. It facilitates mTORC1 interactions with substrates, impacting translation initiation and metabolic processes essential for cellular function [117,178,179]. Also associates with RPS6 and upregulates S6 kinase.	Raptor, as part of mTOR complex 1 (mTORC1), has roles in the brain by regulating protein synthesis, neuronal growth, and synaptic plasticity, oligodendrocyte differentiation, and myelination [97,130]. These processes are essential for learning, memory formation, and cognitive function, with Raptor's dysregulation linked to neurological disorders such as Alzheimer's disease [114].
^{pS792}-Raptor	Serine-phosphorylated Raptor	Phosphorylation by AMP kinase inhibits mTOR pathway [180].	Inhibits mTORC1 signaling and functions as a metabolic checkpoint set by stress states such as nutrient deprivation [181].
Rictor	Rapamycin-insensitive companion of mTOR	Rictor, a key component of mTOR complex 2 (mTORC2), regulates cell proliferation, survival, and metabolism by activating AKT through phosphorylation at Ser473. It also modulates phosphorylation of Protein Kinase C-alpha and the actin cytoskeleton organization [182].	Rictor influences neuronal survival, cytoskeletal organization, and glucose metabolism. Its activity supports synaptic plasticity, neuroprotection, energy metabolism, and overall brain health [183], with its dysfunction associated with cognitive impairments and neurodegenerative diseases . Rictor ablation or depletion causes microcephaly, alters neurite organization, and impairs motor function [184].

^{pS1591}-Rictor	Serine-phosphorylated Rictor	Phosphorylated by mTOR when part of mTORC2 complex. Also phosphorylated by S6K1 (p70S6K). Phosphorylation inhibits mTORC2 and Akt signaling [185].	Modulates mTORC 2 activity in the brain, may be abnormal in Alzheimer's disease [166,185].
